# Pharmaceutical Salts of Piroxicam and Meloxicam with Organic Counterions

**DOI:** 10.1021/acs.cgd.2c00722

**Published:** 2022-10-21

**Authors:** Shan Huang, Dean S. Venables, Simon E. Lawrence

**Affiliations:** † School of Chemistry, Analytical and Biological Chemistry Research Facility, Synthesis and Solid State Pharmaceutical Centre, University College Cork, Cork T12 K8AF, Ireland; ‡ School of Chemistry and Environmental Research Institute, University College Cork, Cork T12 K8AF, Ireland

## Abstract

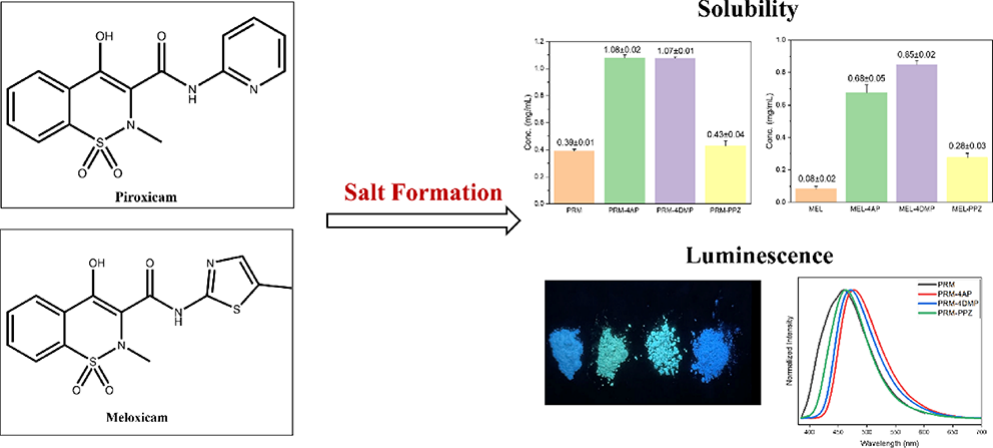

Piroxicam (PRM) and meloxicam (MEL) are two nonsteroidal anti-inflammatory drugs, belonging to the Biopharmaceutics Classification System Class II drugs. In this study, six novel pharmaceutical salts of PRM and MEL with three basic organic counterions, that is, 4-aminopyridine (4AP), 4-dimethylaminopyridine (4DMP), and piperazine (PPZ), were prepared by both slurrying and slow evaporation. These salts were characterized by single-crystal and powder X-ray diffraction, thermal analysis, and Fourier transform infrared spectroscopy. All six salts, especially MEL-4DMP and MEL-4AP, showed a significantly improved apparent solubility and dissolution rate in sodium phosphate solution compared with the pure APIs. Notably, PRM-4AP and PRM-4DMP salts exhibited enhanced fluorescence, and the PRM-PPZ salt showed weaker fluorescence compared with that of pure PRM due to different luminescence mechanisms.

## Abstract 2

Six novel pharmaceutical salts of piroxicam and meloxicam with three basic organic counterions were synthesized. All salts showed a significantly improved apparent solubility and dissolution rate in sodium phosphate solution compared with the pure APIs. Notably, piroxicam salts exhibited different luminescent properties compared with pure piroxicam due to different luminescence mechanisms.

## Introduction

Over the past few decades, the wide application of high-throughput screening in drug design and discovery has resulted in more drug candidates exhibiting low aqueous solubility. Many approaches, including the use of nanoparticles,^1^ lipid-based drug delivery systems^2^ and cyclodextrin inclusion techniques,^3^ have been successfully developed to enhance the solubility of active pharmaceutical ingredients (APIs). For ionizable (acidic, basic, and zwitterionic) APIs, salt formation is the most commonly used method for enhancing aqueous solubility.^4−6^ To some extent, this approach can produce predictable and designable physicochemical properties and performance of drug substances.^7−11^ Approximately 50% of all drug molecules present in marketed products are administered as salts,^12,13^ for example, Zontivity (vorapaxar sulfate), Kisqali (ribociclib succinate), and Ofev (nintedanib esylate). For the preparation of pharmaceutical salts, Δp*K*_a_ is one of the most important factors when considering salt formation, and salts can be distinguished from cocrystals by the degree of proton transfer between the components. Generally, systems with Δp*K*_a_ < 0 lead to cocrystals, and Δp*K*_a_ > 3 results in salts, while 0 < Δp*K*_a_ < 3 can form either of them [where Δp*K*_a_ = p*K*_a_ (base) – p*K*_a_ (acid)].^14^ In addition, complementary hydrogen bond acceptors/donors should exist in the structures of the drug molecule and salt formers.^15^ It is also important that the salt formers should be pharmaceutically acceptable or on the Generally Recognized as Safe (GRAS) and the European Food Safety Authority (EFSA) lists to ensure that the resulting pharmaceutical salts are safe. Based on the presence of acidic or basic functional groups in ionizable APIs, potential counterions can be selected. For basic drugs, chloride and sulfate are typically the most popular inorganic counterions, while acidic drugs usually form salts with simple inorganic cations such as sodium, magnesium, and potassium.^16^ Recently, pharmaceutically acceptable organic counterions have received increasing attention because they tend to have preferable dissolution behaviors. There is no common ion effect in gastric media for the systems compared to the hydrochloride salts, and salt disproportionation may be reduced owing to the regulation of the microenvironmental pH by the organic counterions.^17,18^

**Figure 1:**
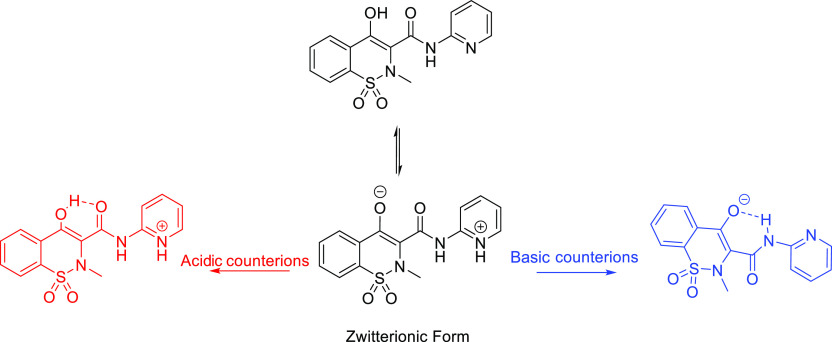
Neutral, zwitterionic (middle), acidic (left), and basic (right) salt forms of PRM.

**Figure 2:**
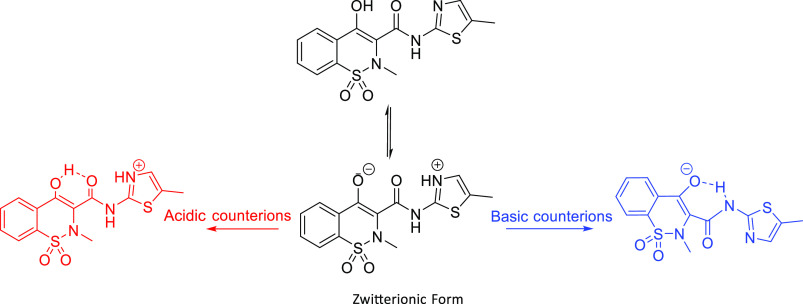
Neutral, zwitterionic (middle), acidic (left), and basic (right) salt forms of MEL.

Piroxicam (PRM, brand name Feldene, Figure 1) and meloxicam (MEL, brand name Mobic, Figure 2) are nonsteroidal anti-inflammatory drugs, prescribed for the symptomatic relief of rheumatoid arthritis and osteoarthritis, which belong to the Biopharmaceutics Classification System Class II drugs, and thus, the bioavailability is considered to be dissolution rate limited.^19,20^ Moreover, PRM and MEL are zwitterionic compounds with p*K*_a_ values of p*K*_a1_ = 1.86 (hydroxyl group) and p*K*_a2_ = 5.46 (pyridyl group)^21^ and p*K*_a1_ = 1.09 (hydroxyl group) and p*K*_a2_ = 4.18 (nitrogen of the 5-methyl-1,3-thiazolyl group),^22^ respectively. When forming salts with acid counterions, the nitrogen atom of the pyridine ring or the thiazole ring from PRM or MEL is deprotonated, whereas when basic counterions are involved in the salt formation, the phenolic hydroxyl group is deprotonated (Figures 1 and 2).

Both PRM and MEL are polymorphic. PRM has six polymorphs (forms I, α1, II, III, VI, and VII),^23^ and it exists as the zwitterionic form in its monohydrate,^24^ while five polymorphs (forms I, II, III, IV, and V) of MEL have been reported,^25^ and the zwitterionic form of MEL in the solid form can be found in form IV^26^ and its monohydrate.^27^

Childs and Hardcastle investigated the cocrystal formation of PRM with pharmaceutically acceptable carboxylic acids using a crystal engineering approach.^20^ Wilson et al. demonstrated that the PRM molecule can exist in two possible tautomers when cocrystallizing with mono-substituted benzoic acids.^28^ Subsequently, they synthesized different multicomponent molecular crystals (including cocrystals and salts) of PRM with *N*-heterocycles and haloanilic acids, and significantly enhanced solubility can be found in some crystals.^29^ Zaworotko and co-workers synthesized 12 cocrystal forms of MEL with carboxylic acids via crystal engineering and the supramolecular synthon approach. Solubility tests and pharmacokinetics studies on these MEL cocrystals revealed that 9 out of 12 cocrystals exhibited a greater apparent solubility and higher oral bioavailability compared to that of pure MEL.^19,30^ In addition to PRM and MEL, two more oxicam drugs (lornoxicam, LRM, and tenoxicam, TNM) have had multicomponent forms synthesized and their physicochemical properties investigated. Nangia et al. synthesized a series of cocrystals and salts of LRM and TNM, indicating the solubility advantages of those new multicomponent crystalline forms.^31,32^ The known multicomponent forms of these four oxicam drugs are shown in Table 1.

**Table 1:** Summary of Salts and Cocrystals and the Physicochemical Properties of PRM, MEL, LRM, and TNM

name	system	multicomponent formers	physicochemical properties^a^
PRM	salt/salt solvate	4-aminopyridine, 4-dimethylaminopyridine, piperazine (this work)	higher solubility at pH 6.5, modified luminescence
		l-arginine^b^^,^^33^	higher solubility in H_2_O, greater bioavailability (x 1.38)
		ethanolamine,^b^ diethanolamine,^b^ triethanolamine^b^^21^	no improvement in solubility at pH 1.2, higher bioavailability and solubility at pH 6.8
		norfloxacin MeOH^34^	higher solubility at pH 6.8 (x 1.4)
	cocrystal/cocrystal solvate	benzoic acid^35^	increased solubility (x 3), increased dissolution rate in H_2_O (x 2) improved oral bioavailability in rats (x 15)
		clonixin ethyl acetate^36^	improved moisture stability
		febuxostat^37^	improved dissolution at pH 6.8 (x 2.8), improved flow and compressibility
		ferulic acid^38^	improved IDR at pH 2 (x 1.7), improved powder flowability
		furosemide^39^	good thermal stability, good stability under accelerated aging
		nicotinamide,^b^ resorcinol,^b^ saccharin sodium,^b^ urea^b^^,^^40^	no solubility advantage
		methylparaben,^b^ vanillin^b^^,^^41^	no solubility and IDR advantages at pH 1.2, superior dissolution rates in the sink condition at pH 1.2
		saccharin^42^	reduced plasticity and significantly deteriorated tableting behavior
		sodium acetate^b^^,^^40^	improved solubility (x 5), improved flow and compressibility
MEL	salt/salt solvate	4-aminopyridine, 4-dimethylaminopyridine, piperazine (this work)	higher solubility at pH 6.5
		arginine^b^^,^^43,44^	improved dissolution behavior at pH 1.2 (x 9.4) and 7.5
		ciprofloxacin MeCN^34^	higher solubility at pH 6.8 (x 3)
		cysteine,^b^ glycine^b^^,^^43^	improved dissolution behavior at pH 7.5
		meglumine^b^^,^^45^	improved solubility at pH 6
		KOH H_2_O^b^^,^^46^	improved dissolution behavior at pH 5.6, no bioavailability advantage in vivo
		di-/triethanolamine,^b^ tris(hydroxymethyl)aminomethane,^b^ KOH^b^^,^^44^	improved dissolution behaviors at pH 1.2 (x 3.7–7.2)
	salt cocrystal	l-malic acid^19,30^	no solubility advantage at pH 6.5, improved bioavailability (x 1.2)
	cocrystal/cocrystal solvate	adipic acid^30,47^	no solubility advantage at pH 6.8
		Aspirin^48^	improved solubility at pH 7.4 (x 44), improved bioavailability (x 4.4)
		benzoic acid,^19,49^ 4-hydroxybenzoic acid,^b^^,^^19,30^ 1-hydroxy-2-naphthoic acid,^19,30^dl-malic acid,^b^^,^^19,30,50^ salicylic acid,^19^ succinic acid^19,30,47^	higher solubility at pH 6.5, improved bioavailability (x 1.1–1.6)
		(+)-camphoric acid^b^^,^^30^	no solubility advantage at pH 6.5
		fumaric acid^19,30,50^	higher solubility at pH 6.5 and 6.7, no bioavailability advantage
		glutaric acid^19,30^	no solubility advantage at pH 6.5, improved bioavailability (x 1.2)
		glycolic acid^b^^,^^19,30^	no solubility advantage at pH 6.5, no bioavailability advantage
		hydrocinnamic acid^b^^,^^19,30^	higher solubility at pH 6.5, no bioavailability advantage
		maleic acid^b^^,^^19,30,51^	higher solubility at pH 1.6, 5.0, and 6.5, improved bioavailability (x 1.2)
		salicylic acid^30,50−52^	higher solubility at pH 1.6, 5.0, and 6.5, enhanced drug permeation coefficient
		terephthalic acid^47^	higher solubility at pH 6.8
LRM	salt/salt solvate	HCl, methanesulfonic acid, NH_3_, piperazine,^31^	improved dissolution at pH 7 (x 1.3–1.6)
		norfloxacin H_2_O MeOH^34^	improved dissolution at pH 6.8 (x 1.6)
	cocrystal/cocrystal solvate	ascorbic acid,^b^ benzoic acid,^b^ cinnamic acid,^b^ citric acid,^b^ fumaric acid,^b^ glutaric acid,^b^ hippuric acid,^b^ malonic acid,^b^ salicylic acid,^b^ succinic acid,^b^ tartaric acid^b^^,^^53^	no solubility advantage in H_2_O
		4-aminobenzoic acid,^b^ anthranilic acid,^b^ ferulic acid,^b^ 4-hydroxy benzoic acid,^b^ oxalic acid,^b^ resorcinol,^b^ saccharin sodium,^b^ urea^b^^,^^53^	improved solubility in H_2_O (x 1.6–6.9)
		1,3-dimethyl urea^b^,^54^	increased IDR at pH 1.2 (x 28) and 7.4 (x 19), improved tabletability (x 2.5) and bioavailability(x 2.5)
TNM	salt/salt solvate	ciprofloxacin MeOH^34^	improved dissolution at pH 6.8 (x 1.1)
		HCl, methanesulfonic acid^32^	no solubility and IDR advantages at pH 7
		piperazine^32^	improved solubility (x 5.5) and IDR (x 2.5) at pH 7
	cocrystal/cocrystal solvate	benzoic acid^32^	no solubility advantage, improved IDR (x 2) at pH 7
		catechol, pyrogallol, resorcinol^32^	improved solubility (x 5.8–10.1) and IDR(x 2.4–4.2) at pH 7
		glycolic acid,^b^ saccharin,^b^ salicylic acid,^b^ succinic acid,^b^^,^^55^	no IDR advantage at pH 4.5 and 6.8
		salicylic acid^32^	no solubility and IDR advantages at pH 7

a No reported physicochemical properties of multicomponent forms for PRM,^20,28,36,56,57^ MEL,^27,30,58^ and TNM.^55^

b No crystal structure reported.

It is known that PRM exhibits luminescence in solution,^59^ and there has been recent interest in the solid-state luminescent properties of organic multicomponent crystalline materials, with applications including organic light-emitting diodes, semiconductor lasers, and fluorescent sensors.^60−63^

In many conventional systems, fluorophores that exhibit intensive fluorescence in the solution state can experience partial or complete emission quenching in the aggregate state. This is due to the effects of excited-state energy transfer in the solid state and is known as aggregation-caused quenching (ACQ).^64−66^ In 2001, Tang’s group reported that silole derivatives exhibited significantly enhanced fluorescence in the aggregated state and proposed the concept of aggregation-induced emission to explain this phenomenon.^67^ Recent years have witnessed the wide application of solid-state fluorescence in the pharmaceutical, food, chemical, and optoelectronic industries. For example, Tang’s group developed a real-time, on-site, and nondestructive fluorescence imaging technique to monitor the crystal formation and transformation based on the crystallization-induced emission properties of (*Z*)-1-phenyl-2-(3-phenylquinoxalin-2(1*H*)-ylidene)ethenone. Based on the in-depth analysis of the crystal structures of two crystalline polymorphs, Li et al. demonstrated that the mechanoluminescence performance is related to the molecular packing rather than the chemical structure.^68^

To date, the reports of cocrystals/salts of PRM or MEL with organic basic cocrystal/salt formers are rare, and the solid-state luminescent properties of PRM have not been reported. Therefore, one motivation for this study is to explore whether the crystal landscape of PRM and MEL can be expanded with more bases and, if so, is there any improvement in solubility. Another motivation is to investigate the solid-state luminescence behavior of PRM and its salts and expand the understanding of crystal engineering in modifying the luminescent properties of organic materials.

**Figure 3:**
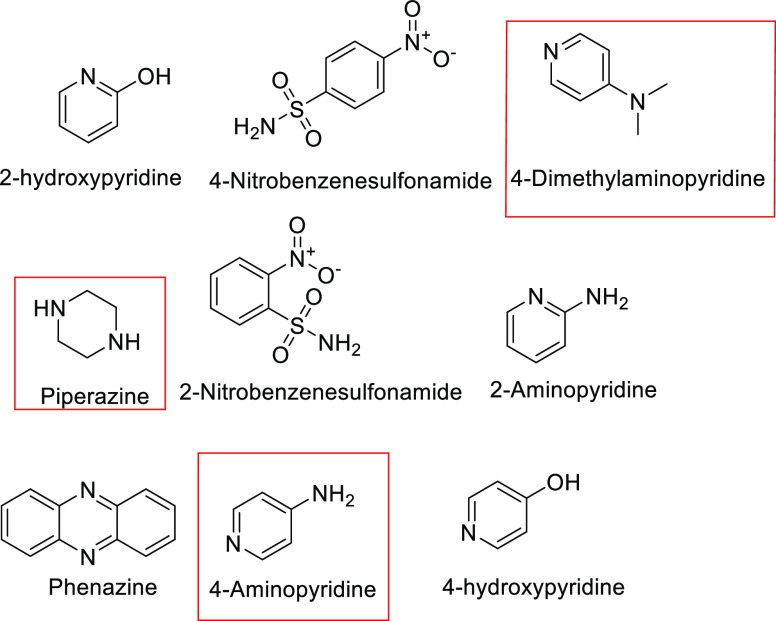
Molecular structures of the salt formers used in this study (the red boxes indicate those compounds that successfully formed salts with PRM and MEL).

In this work, six novel pharmaceutical salts of PRM or MEL with 4-aminopyridine (4AP), 4-dimethylaminopyridine (4DMP), or piperazine (PPZ) (Figure 3) were prepared and characterized by various solid-state analytical techniques, including thermal analysis, X-ray techniques, and Fourier transform infrared (FT-IR) spectroscopy. The solubility behavior of the six salts was measured and compared with those of parent materials. The distinct luminescent properties of PRM and its salts were investigated by optical-physical techniques together with the Hirshfeld surface analysis and the frontier molecular orbital (FMO) analysis.

## Experimental Section

### Materials

PRM (form I) and MEL (form I) were purchased from Fluorochem and used as received without further purification. All salt formers were obtained from Sigma-Aldrich and used as received. Solvents were purchased from commercial sources and used as received.

### Synthesis of Salts

#### PRM-4AP Salt

PRM (49.7 mg, 0.15 mmol) and 4AP (14.1 mg, 0.15 mmol) in a 1:1 molar ratio were dissolved in 5 mL of methanol by heating. Red plate-like crystals were obtained by slowly evaporating the filtrated solution for 3 days. Bulk materials were made by slurrying a stoichiometric amount (1:1) of PRM (331.5 mg, 1 mmol) and 4AP (94.1 mg, 1 mmol) in 3 mL of methanol at room temperature for 3 days. The resulting suspension was allowed to dry in the fume hood. The powdered product was isolated and analyzed by powder X-ray diffraction (PXRD).

#### PRM-4DMP Salt

PRM (49.7 mg, 0.15 mmol) and 4DMP (18.3 mg, 0.15 mmol) in a 1:1 molar ratio were dissolved in 10 mL of acetone by heating. Yellow plate-like crystals were obtained by slowly evaporating the filtrated solution for 3 days. Bulk materials were made by slurrying a stoichiometric amount (1:1) of PRM (331.5 mg, 1 mmol) and 4DMP (122.2 mg, 1 mmol) in 3 mL of methanol at room temperature for 3 days. The resulting suspension was allowed to dry in the fume hood. The powdered product was isolated and analyzed by PXRD.

#### PRM-PPZ Salt

PRM (49.7 mg, 0.15 mmol) and PPZ (6.5 mg, 0.075 mmol) in a 2:1 molar ratio were dissolved in 5 mL of nitromethane by heating. Yellow needle-like crystals were obtained by slowly evaporating the filtrated solution for 5–8 days. Bulk materials were made by slurrying a stoichiometric amount (2:1) of PRM (331.5 mg, 1 mmol) and PPZ (43.1 mg, 0.5 mmol) in 3 mL of methanol at room temperature for 3 days. The resulting suspension was allowed to dry in the fume hood. The powdered product was isolated and analyzed by PXRD.

#### MEL-4AP Salt

MEL (52.7 mg, 0.15 mmol) and 4AP (14.1 mg, 0.15 mmol) in a 1:1 molar ratio were dissolved in 10 mL of acetone by heating. Yellow needle-like crystals were obtained by slowly evaporating the filtrated solution for 5–8 days. Bulk materials were made by slurrying a stoichiometric amount (1:1) of MEL (351.4 mg, 1 mmol) and 4AP (94.1 mg, 1 mmol) in 3 mL of acetone at room temperature for 3 days. The resulting suspension was allowed to dry in the fume hood. The powdered product was isolated and analyzed by PXRD.

#### MEL-4DMP Salt

MEL (52.7 mg, 0.15 mmol) and 4DMP (18.3 mg, 0.15 mmol) in a 1:1 molar ratio were dissolved in 5 mL of methanol by heating. Yellow plate-like crystals were obtained by slowly evaporating the filtrated solution for 8–10 days. Bulk materials were made by slurrying a stoichiometric amount (1:1) of MEL (351.4 mg, 1 mmol) and 4DMP (122.2 mg, 1 mmol) in 3 mL of methanol at room temperature for 3 days. The resulting suspension was allowed to dry in the fume hood. The powdered product was isolated and analyzed by PXRD.

#### MEL-PPZ Salt

MEL (52.7 mg, 0.15 mmol) and PPZ (6.5 mg, 0.075 mmol) in a 2:1 molar ratio were dissolved in 10 mL of DMF–EtOAc (1:1, v/v) by heating. Yellow needle-like crystals were obtained by slowly evaporating the filtrated solution for 5–8 days. Bulk materials were made by slurrying a stoichiometric amount (2:1) of MEL (351.4 mg, 1 mmol) and PPZ (43.1 mg, 0.5 mmol) in 3 mL of methanol at room temperature for 3 days. The resulting suspension was allowed to dry in the fume hood. The powdered product was isolated and analyzed by PXRD.

### Physical Measurements

Differential scanning calorimetry (DSC) data were collected using a TA Instruments Q1000. Samples (2–6 mg) were crimped in nonhermetic aluminum pans and scanned from 25 to 300 °C at a heating rate of 10 °C min^–1^ under a continuously purged dry nitrogen atmosphere. Thermogravimetric analysis (TGA) data were collected using a TA Instruments Q500 thermogravimetric analyzer. The sample was placed in an aluminum sample pan and heated under nitrogen at a rate of 20 °C min^–1^ from 25 to 500 °C. IR spectra were recorded on a PerkinElmer UATR Two spectrophotometer using a diamond attenuated total reflectance accessory over a range of 400–4000 cm^–1^. An average of four scans was taken for each spectrum obtained with a resolution of 4 cm^–1^. PXRD data were collected using a STOE STADI MP diffractometer with Cu Kα radiation using a linear position-sensitive detector (PSD) over the 2θ range of 3.5–45.5° with an increment of 0.05° at a rate of 2° min^–1^. The samples were prepared as transmission foils, and the data were viewed via STOE WinXPOW POWDAT software.^69^ Single-crystal XRD (SCXRD) data were collected on a Bruker APEX II DUO with monochromated Cu Kα radiation for PRM-4AP and MEL-4DMP (λ = 1.54184 Å) and Mo Kα radiation for PRM-4DMP, PRM-PPZ, MEL-4AP, and MEL-PPZ (λ = 0.7107 Å), respectively. All calculations and refinements were made using Bruker APEX software with the SHELXL program.^70,71^ Nonhydrogen atoms were refined anisotropically. All hydrogen atoms were placed in geometrically calculated positions using the riding model, with C–H = 0.93–0.97 Å and N–H = 0.86–0.89 Å and *U*_iso_ (H) (in the range 1.2–1.5 times *U*_eq_ of the parent atom). For PRM-PPZ and MEL-PPZ, there was disorder in the methyl group, which was modeled in two conformations in a 50:50 ratio. DIAMOND was used for creating figures,^62^ and PLATON was used for the analysis of potential hydrogen bonds and short-ring interactions.^72^ Crystallographic parameters are listed in Table 2.

**Table 2:** Crystallographic Data for PRM-4AP, PRM-4DMP, PRM-PPZ, MEL-4AP, MEL-4DMP, and MEL-PPZ Salts

	PRM-4AP 1:1	PRM-4DMP 1:1	PRM-PPZ 1:0.5	MEL-4AP 1:1	MEL-4DMP 1:1	MEL-PPZ 1:0.5
chemical formula	C_20_H_19_N_5_O_4_S	C_22_H_23_N_5_O_4_S	C_17_H_18_N_4_O_4_S	C_19_H_19_N_5_O_4_S_2_	C_21_H_23_N_5_O_4_S_2_	C_16_H_18_N_4_O_4_S_2_
formula weight	425.46	453.52	374.41	445.51	473.56	394.46
crystal system	triclinic	monoclinic	monoclinic	monoclinic	triclinic	monoclinic
space group	*P*1̅	*C*2/*c*	*P*2_1_/*c*	*Cc*	*P*1̅	*P*2_1_/*c*
temperature (K)	293(2)	296	299(2)	296	298(2)	298(2)
a (Å)	8.2328(12)	22.431(3)	9.944(4)	11.813(2)	8.3482(7)	8.002(4)
b (Å)	10.6200(16)	16.048(3)	7.906(3)	20.427(4)	10.4251(15)	29.90(2)
c (Å)	12.361(4)	12.900(2)	22.650(12)	10.2167(17)	12.6635(7)	7.512(4)
α (°)	75.393(16)	90	90	90	90.789(7)	90
β (°)	71.368(12)	109.888(6)	96.56(2)	123.016(3)	95.355(5)	106.380(18)
γ (°)	89.872(10)	90	90	90	95.567(7)	90
volume (Å^3^)	987.4(4)	4366.7(13)	1769.1(13)	2067.1(6)	1091.84(19)	1724.6(18)
*Z*	2	8	4	4	2	4
ρ_calc_ (g cm^–3^)	1.431	1.380	1.406	1.432	1.444	1.519
radiation type	Cu Kα	Mo Kα	Mo Kα	Mo Kα	Cu Kα	Mo Kα
μ (mm^–1^)	1.795	0.188	0.214	0.295	2.551	0.340
reflns measured	40074	27903	57483	7186	57119	76271
reflns independent	3809	5504	9923	3309	4267	6931
significant [*I* > 2σ(*I*)]	3670	4248	7423	3236	4010	3067
parameters refined	272	292	235	273	290	235
restraints	0	6	0	26	0	0
Δρ_max_, Δρ_min_ (e Å^–3^)	0.367, −0.465	0.381, −0.451	0.404, −0.417	0.140, −0.232	0.331, −0.559	0.385, −0.445
*F*(000)	444	1904	784	928	498	824
*R*_*1*_ [*I* > 2σ(*I*)]	0.0435	0.0561	0.0425	0.0260	0.0344	0.0609
w*R*_*2*_ (all data)	0.1193	0.1200	0.1338	0.0654	0.1017	0.1916
CCDC number	2064483	2109805	2064485	2109802	2109803	2109804

### Computational Studies

Hirshfeld surface analyses and two-dimensional (2D) fingerprint plots were obtained using the CrystalExplorer 21.5 program.^73^ Density functional theory calculations using the Gaussian 09 program package employing the *RB3LYP* functional with the 6-31G (d, p) basis set were performed on PRM, MEL, and the six obtained crystals without conducting structural optimization.^62,74^ The molecular orbitals were viewed using the Multiwfn 3.8 program and plotted using VMD.^75,76^

### Solubility Experiments

Solubility experiments were conducted in sodium phosphate buffer solutions at pH 6.5 and 37 °C to simulate intestinal physiological conditions. For each experiment, an excess crystalline solid was sieved through a 300 μm sieve and introduced into a flask with a screw top containing 100 mL of the medium. The solution was stirred at 200 rpm using a magnetic stir bar for 48 h to reach the equilibrium state. Sampling was performed at 5, 10, 15, 20, 30, 45, 60, 90, 120, 180, 240, 300, 360, 540, 720, 1440, 2160, and 2880 min. After each sampling, the volume of the liquid removed from the suspension was not compensated. The withdrawn suspension was filtered through 0.2 μm nylon filters and diluted prior to high-performance liquid chromatography (HPLC) analysis. The solubility experiment for each crystal form was repeated in triplicate. After the last sample collection, the remaining solid material in the suspension was filtered, dried, and characterized by IR and PXRD.

The HPLC method was developed to determine the concentration of PRM and MEL using an Agilent 1260 series Infinite HPLC system (Agilent Technologies, Waldbronn, Germany). A C18 HPLC column (YMC-Pack ODS-A column, 4.6 mm × 250 mm, 5 μm) with a flow rate of 1 mL min^–1^ was employed, and the column temperature was set at 25 °C. The binary mobile phase consisted of acetonitrile and sodium phosphate buffer (pH 6.5) in a volume ratio of 25:75. The samples were diluted appropriately with the mobile phase, and the absorbance was measured at 347 nm. The retention times of PRM and MEL were 7.1 min and 10.9 min, respectively.

### Optical-Physical Measurements

Solid-state UV–vis spectra were recorded on a Shimadzu 3200 UV spectrometer. Solid-state fluorescent spectra were collected using a Cary Eclipse fluorescence spectrometer (Agilent, United States) with 365 nm excitation light. The fluorescence quantum yield values were measured using a Hamamatsu Photonics C9920-02G instrument (Hamamatsu Photonics Co., Ltd).

## Results and Discussion

### Crystal Structure Analysis

The salt formation of PRM or MEL can be rationalized by Δp*K*_a_ values. The Δp*K*_a_ values are greater than 3 in all cases since 4AP, 4DMP, and PPZ are strong organic bases (Table 3). Therefore, it is expected that the hydroxyl group of PRM or MEL will be deprotonated and form charge-assisted hydrogen-bonded salts with these organic counterions. Single crystals of the six salts suitable for SCXRD were obtained and their structures determined. Ellipsoid plots of PRM-4AP, PRM-4DMP, and PRM-PPZ are shown in Figure S5 and those of MEL-4AP, MEL-4DMP, and MEL-PPZ are shown in Figure S13. Hydrogen bonds and π–π interaction geometries are shown in Tables S1–S3 for PRM-4AP, PRM-4DMP, and PRM-PPZ salts and in Tables S5–S7 for MEL-4AP, MEL-4DMP, and MEL-PPZ salts.

**Table 3:** **p*K***_**a**_Values of PRM, MEL, and Salt Formers and Their Δp*K*_a_ Values

	p*K*_a_ in water	Δp*K*_a_ for PRM	Δp*K*_a_ for MEL	structure
PRM	1.86,^a^ 5.46^21^			
MEL	1.09,^a^ 4.18^22^			
4AP	9.17^77^	7.31	8.08	1:1 salt
4DMP	9.70^78^	7.84	8.61	1:1 salt
PPZ	9.72^79^	7.86	8.63	2:1 salt

a It is the hydroxyl group that is deprotonated in this work.

### PRM-4AP Salt

**Figure 4:**
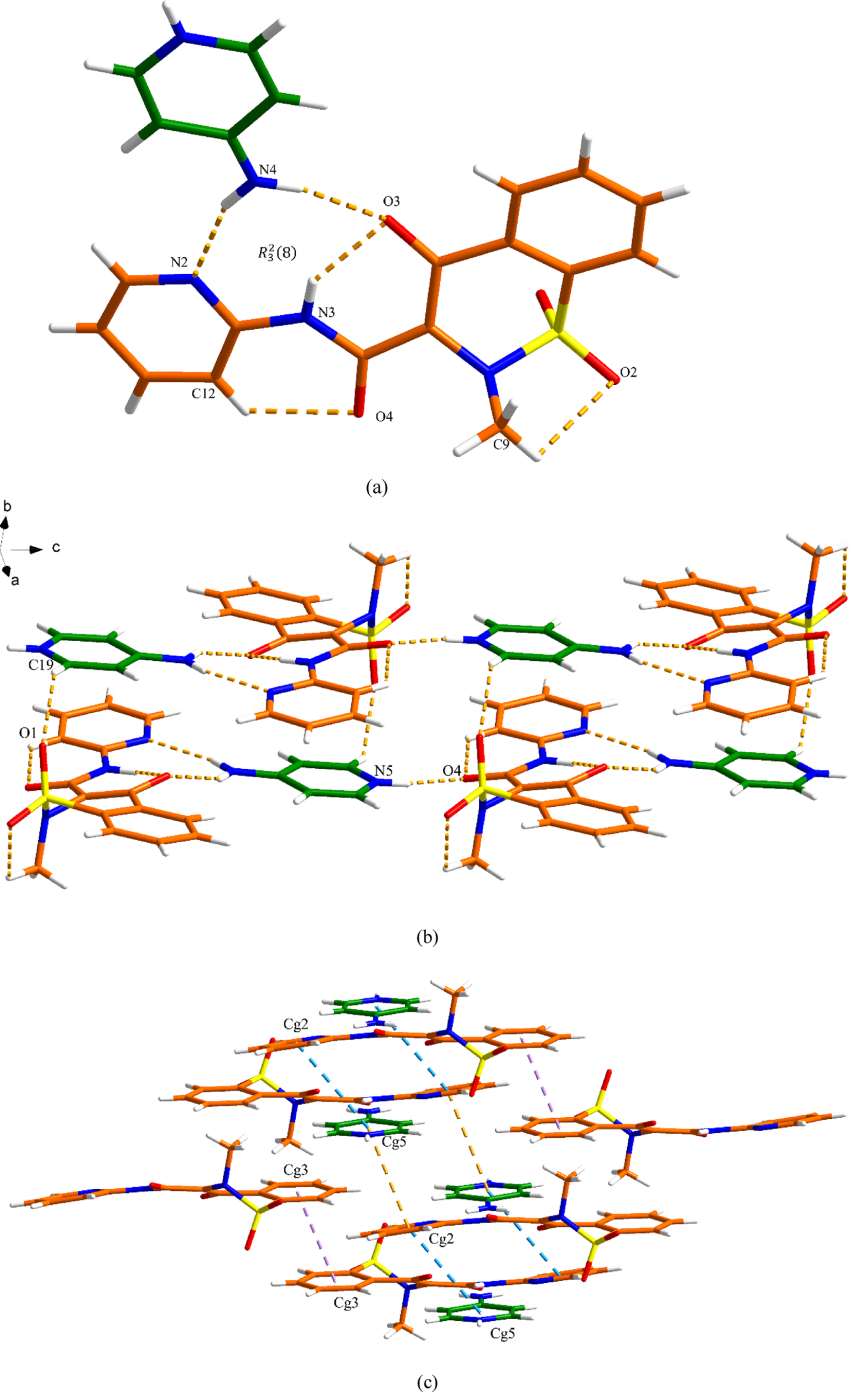
Crystal packing and intermolecular interactions in the PRM-4AP salt: (a) asymmetric unit (orange is PRM and green is 4AP), (b) 2D hydrogen-bonded network (hydrogen bonding is displayed by dashed lines), and (c) 3D network resulting from π–π interactions as indicated by dashed lines (hydrogen bonding is not displayed for clarity).

PRM-4AP crystallizes in the *P*1̅ space group with *Z* = 2, the asymmetric unit consisting of one PRM^–^ anion and one 4-aminopyridinium (4APH^+^) cation. As shown in Figure 4a, a *R*_3_^2^(8) motif is formed between PRM^–^ and 4APH^+^ through two discrete hydrogen bonds (N4–H4A···N2, 2.99 Å, and N4–H4B···O3, 2.89 Å) and intramolecular hydrogen bonding interactions [S(6), N3–H3N···O3, 2.65 Å]. There are two more intramolecular interactions within the PRM^–^ anion (C12–H12···O4, 2.87 Å, and C9–H9B···O2, 2.85 Å) forming a S(6) motif and a S(5) motif, respectively. Notice that the bond angle of the methyl C–H···O intramolecular interaction is smaller than that of other interactions, which is reasonable since five-membered ring intramolecular hydrogen bonds usually have the smallest angles and the longest distances compared to the six-eight-membered intramolecular hydrogen bonds and are within the geometric limits of a hydrogen bond as defined.^80^ Moreover, the angle (τ) formed between the methyl hydrogen and the plane of a sp^2^ oxygen in the PRM^–^ anion is 45.4°, which falls in the required range (<50°).^81^ This methyl C–H···O intramolecular interaction in the PRM^–^ anion can also be found in PRM-4DMP and PRM-PPZ salts, and their dihedral angles (τ) are listed in Figure S6. The basic unit is extended via two discrete hydrogen bonding interactions, that is, N5–H5N···O4 (2.65 Å) and C19–H19···O1 (3.28 Å), resulting in a double-layer 2D network (Figure 4b). As shown in Figure 4c, the π–π interactions between layers participate in the construction of the three-dimensional (3D) structure. The centroid–centroid distances of π–π interactions from Cg2 to Cg5 (orange), Cg5 to Cg2 (blue), and Cg3 to Cg3 (purple) are 4.18, 4.17, and 3.64 Å, respectively (Table S1).

### PRM-4DMP Salt

**Figure 5:**
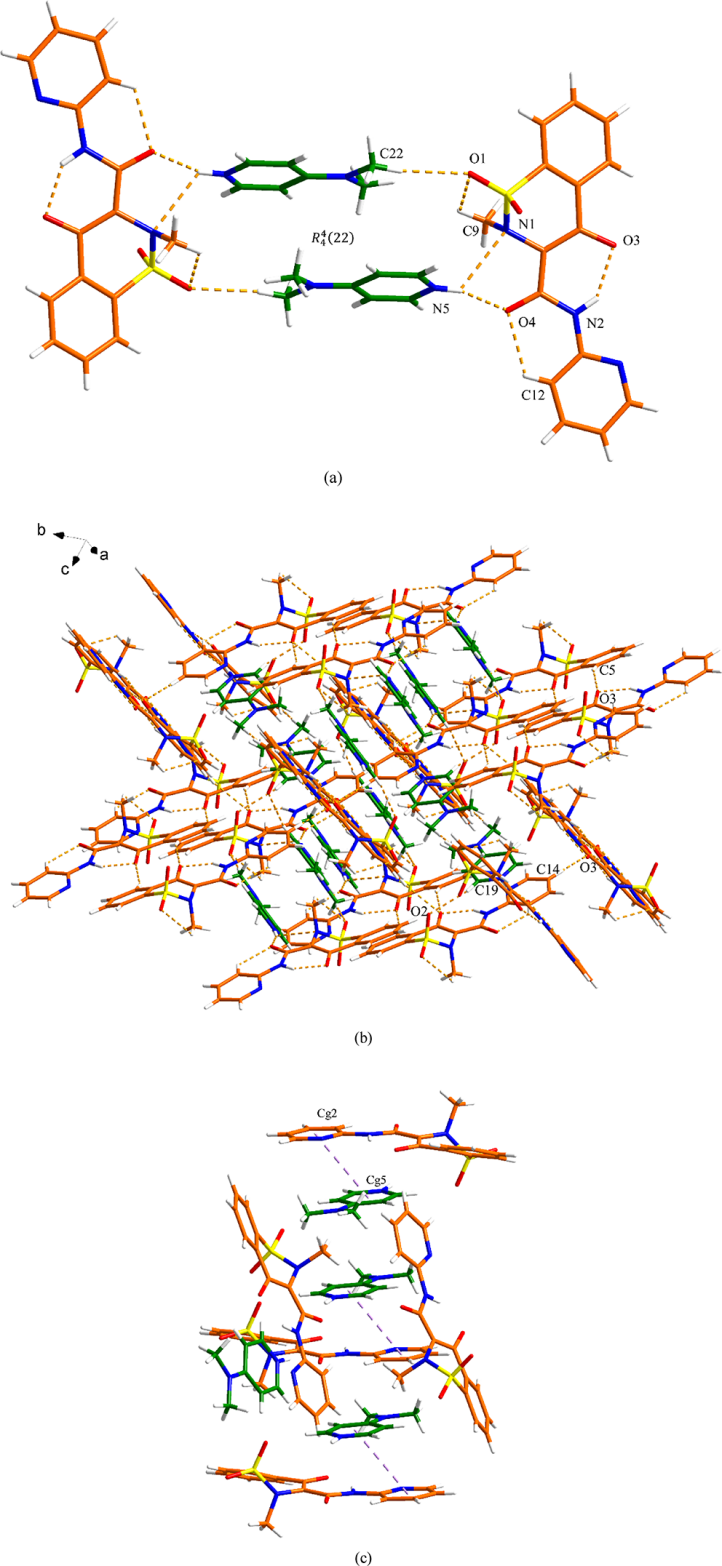
Crystal packing and intermolecular interactions in the PRM-4DMP salt: (a) tetramer (orange is PRM and green is 4DMP), (b) 3D hydrogen-bonded network (hydrogen bonding is displayed by dashed lines), and (c) 3D network resulting from π–π interactions as indicated by dashed lines (hydrogen bonding is not displayed for clarity).

PRM-4DMP crystallizes in the monoclinic *C*2/*c* space group and contains, in the asymmetric unit, one PRM^–^ anion and one 4DMPH^+^ cation. As shown in Figure 5a, one S(5) and two S(6) can be found in the structure of the PRM^–^ anion. The two PRM^–^ anions and two 4DMPH^+^ cations form a *R*_4_^4^(22) motif around an inversion center via two discrete N5–H24···N1 (3.15 Å) and C22–H29···O1 (3.40 Å) hydrogen bonding interactions. The discrete N5–H24···O4 (2.69 Å) hydrogen bond is also responsible for the construction of the tetramer. This tetramer is then extended by four C–H···O hydrogen bond interactions, displaying the 3D hydrogen bonding network (Figure 5b and Table S2). As shown in Figure 5c and Table S2, the 3D structure is further stabilized by π–π interactions between the two pyridyl rings of PRM and 4DMP (Cg2-Cg5, 4.07 Å).

### PRM-PPZ Salt

**Figure 6:**
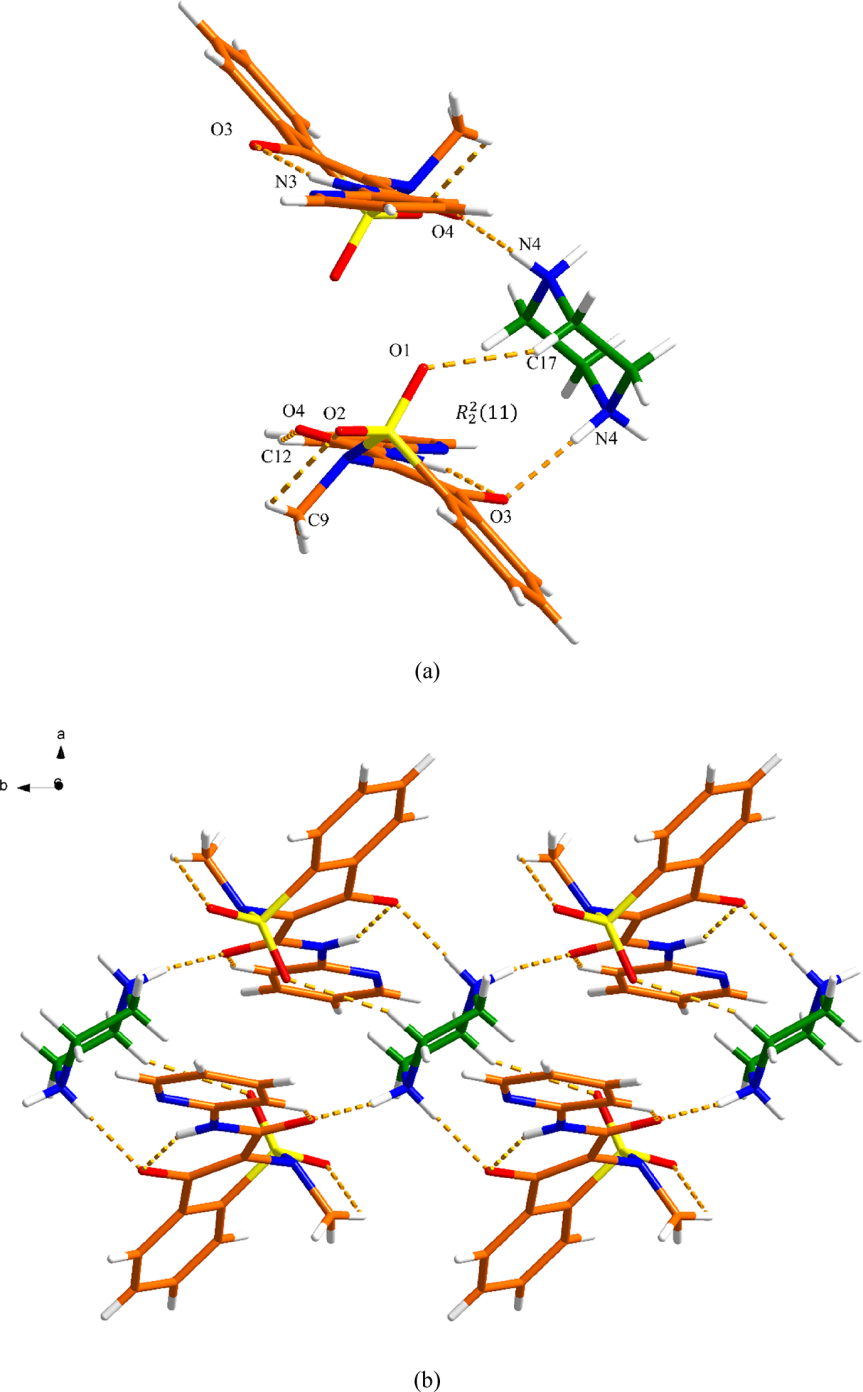
Crystal packing and intermolecular interactions in the PRM-PPZ salt: (a) basic unit (orange is PRM and green is PPZ) and (b) 3D hydrogen-bonded network (hydrogen bonding is displayed by dashed lines). One of the disordered methyl hydrogen atom conformations has been omitted for clarity.

The PRM-PPZ salt crystallizes in the monoclinic system with the space group *P*2_1_/*c* with one PRM^–^ anion and half of the PPZH_2_^2+^ dication in the asymmetric unit. As shown in Figure 6a, the PPZH_2_^2+^ dication is located on an inversion center (protons abstracted each from two PRM molecules), and the two PRM^–^ anions are at the general position in the unit cell. A *R*_2_^2^(11) motif is formed between PPZH_2_^2+^ and PRM^–^ through the discrete N–H···O hydrogen bond interaction (N4–H4B···O3, 2.70 Å) and C–H···O hydrogen bond interaction (C17–H17A···O1, 3.48 Å). Along the *c* axis, there are discrete N–H···O hydrogen bonds (N4–H4A···O4, 2.65 Å) between PPZH_2_^2+^ and another PRM^–^ anion. No significant π–π interactions participate in stabilizing the 3D structure of PRM-PPZ salts (Figure 6b and Table S3).

### MEL-4AP Salt

**Figure 7:**
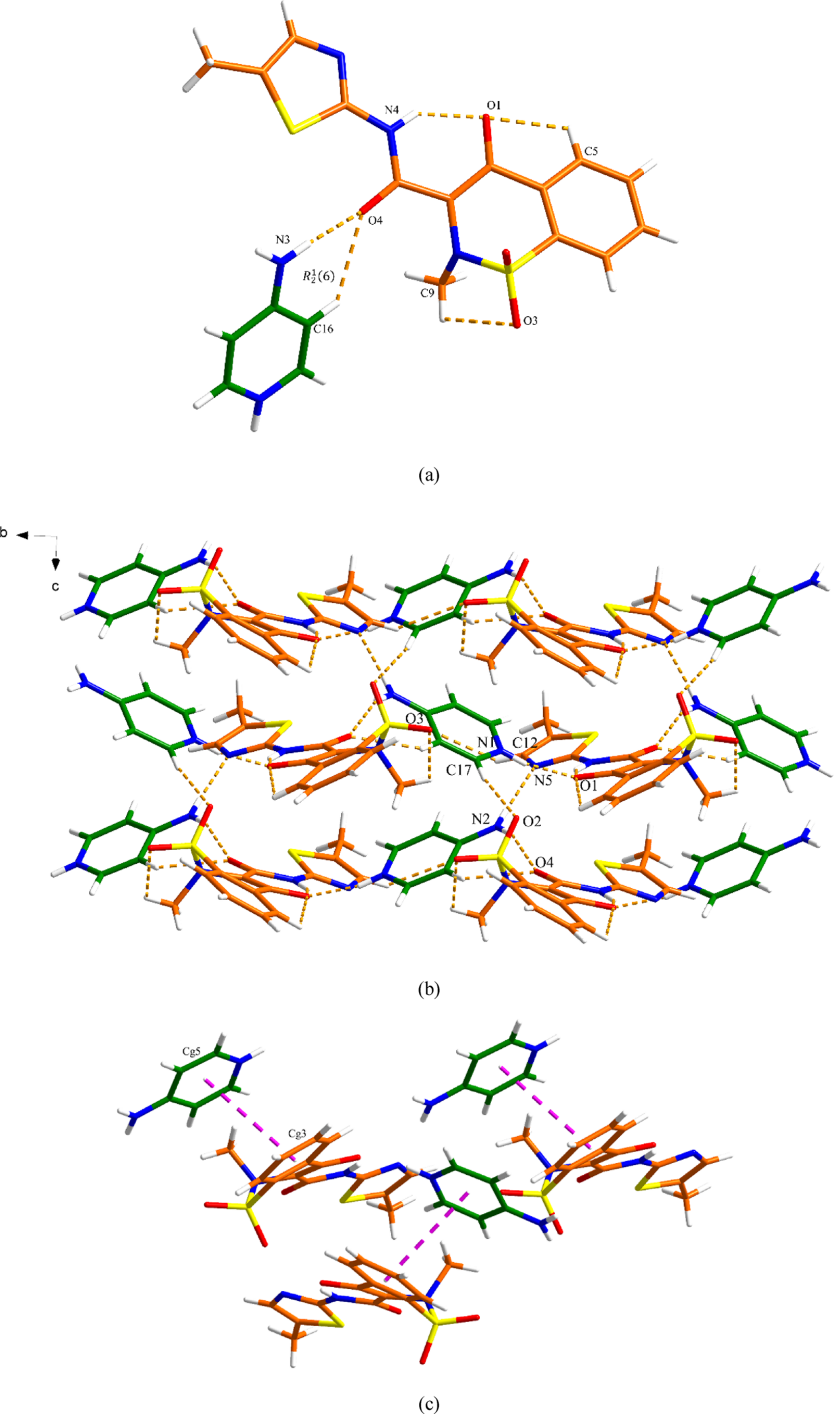
Crystal packing and intermolecular interactions in the MEL-4AP salt: (a) asymmetric unit (orange is MEL and green is 4AP), (b) 3D hydrogen-bonded network (hydrogen bonding is displayed by dashed lines), and (c) 3D network resulting from π–π interactions as indicated by dashed lines (hydrogen bonding is not displayed for clarity).

MEL-4AP salt crystallizes as a 1:1 salt, consistent with the acid–base donor–acceptor ratio. One MEL^–^ anion and one 4APH^+^ cation are present in the asymmetric unit of a monoclinic *Cc* crystal structure. As shown in Figure 7a, a *R*_2_^1^(6) supramolecular heterosynthon is formed between one 4APH^+^ and one MEL^–^ via N–H···O and C–H···O discrete hydrogen bond interactions (N2–H20···O4, 2.89 Å; C16–H16···O, 3.27 Å). The methyl C–H···O intramolecular interaction can be observed in three MEL salts, and their dihedral angles (τ) are listed in Figure S14. Along the *a* axis, the MEL^–^ anion in this unit is further connected with other MEL^–^ and 4APH^+^ through C12–H12···O3 (3.26 Å) and N1–H1···O1 (2.69 Å) hydrogen bonds, respectively, extending into a 2D sheet. These 2D sheets are further linked through N2–H21···N5 (3.02 Å) and C17–H17···O2 (3.29 Å) hydrogen bonds to construct a 3D network, as shown in Figure 7b. The structure is also stabilized by intermolecular π–π stacking interactions between the phenyl ring of MEL and the pyridyl ring of 4AP with a centroid–centroid distance (Cg–Cg) of around 4.22 Å (Figure 7c, Table S5).

### MEL-4DMP Salt

**Figure 8:**
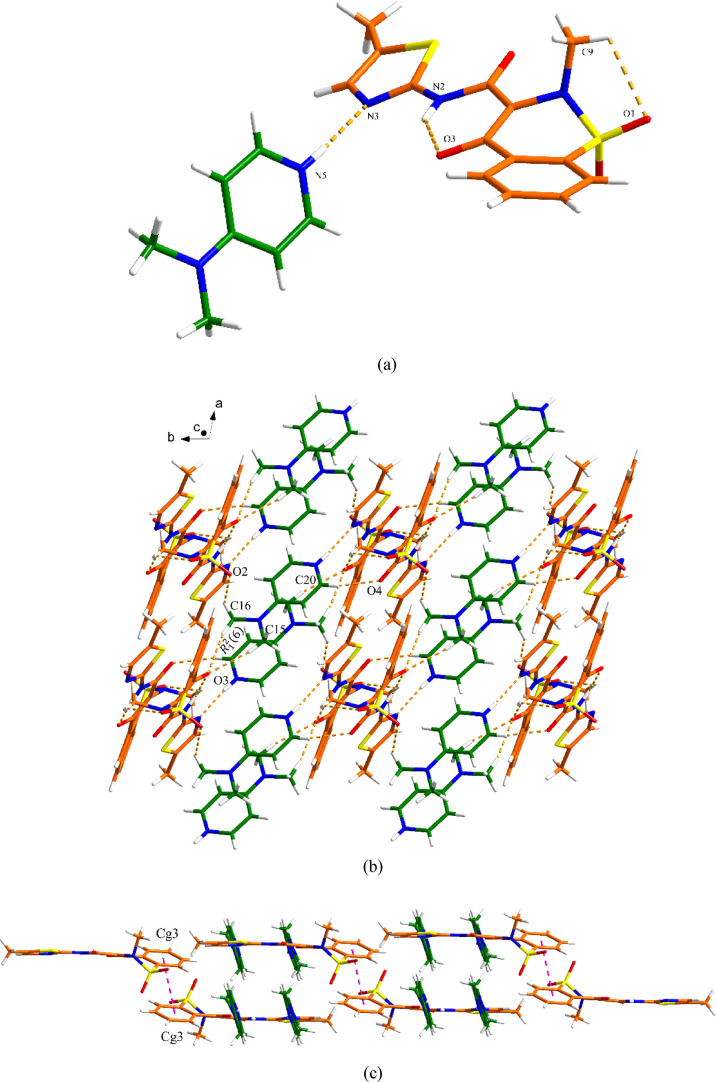
Crystal packing and intermolecular interactions in the MEL-4DMP salt: (a) asymmetric unit (orange is MEL and green is 4DMP), (b) 3D hydrogen-bonded network (hydrogen bonding is displayed by dashed lines), and (c) 3D network resulting from π–π interactions as indicated by dashed lines (hydrogen bonding is not displayed for clarity).

The MEL-4DMP salt crystallizes in the *P*1̅ space group. The asymmetric unit contains one MEL^–^ anion and 4DMPH^+^ cation (Figure 8a). The two components interact via N-H···N (N5-H05···N3, 2.79 Å) discrete hydrogen bond interactions. The 3D structure is further assembled by the C-H···O (C15–H15A···O3, 3.35 Å; C16–H16A···O3, 3.45 Å; C16–H16C···O2, 3.24 Å and C20–H20···O4, 3.16 Å) hydrogen bond interactions between the 4DMPH^+^ cation and adjacent MEL^–^ anions. Additional π–π interactions between the phenyl rings from MEL^–^ (Cg3-Cg3, 3.80 Å) contribute to the extended 3D structure (Figure 8b,c and Table S6).

### MEL-PPZ Salt

**Figure 9:**
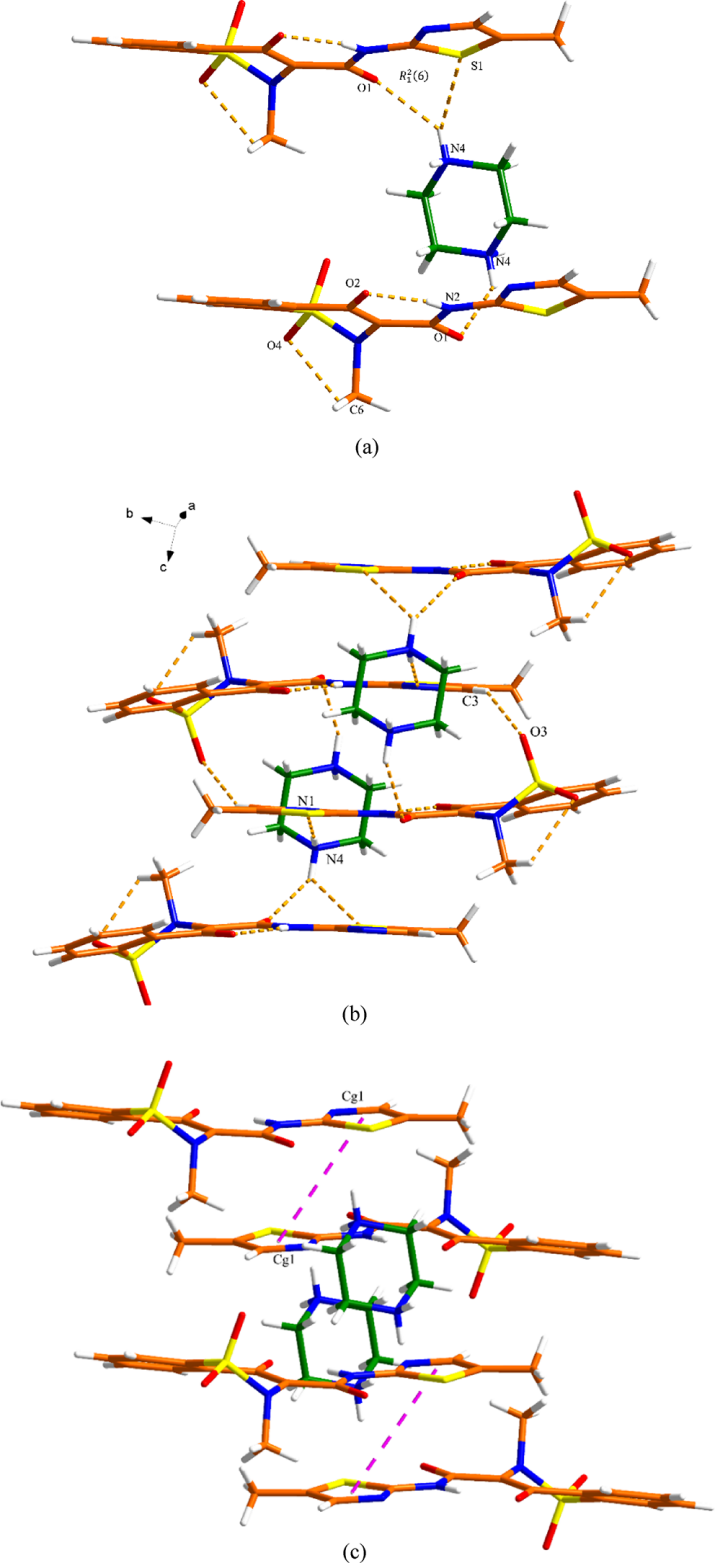
Crystal packing and intermolecular interactions in the MEL-PPZ salt: (a) basic unit (orange is MEL and green is PPZ), (b) 3D hydrogen-bonded network (hydrogen bonding is displayed by dashed lines), and (c) 3D network resulting from π–π interactions as indicated by dashed lines (hydrogen bonding is not displayed for clarity). One of the disordered methyl hydrogen atom conformations has been omitted for clarity.

The MEL-PPZ salt crystallizes in the monoclinic *P*2_1_/*c* space group. The asymmetric unit contains one MEL^–^ anion and half of the PPZH_2_^2+^ dication. As shown in the top of Figure 9a, the PPZH_2_^2+^ dication connects to two MEL^–^ anions via N–H···O and N–H···S (N4–H4B···O1, 2.93 Å; N4–H4B···S1, 3.31 Å) discrete hydrogen bond interactions, generating a *R*_1_^2^(6) motif (as shown in the top of Figure 9a), and N4–H4B···O1 (2.93 Å) discrete hydrogen bonding interactions (in the bottom of Figure 9a). This trimer is connected with adjacent MEL^–^ and PPZH_2_^2+^ via N–H···N and C–H···O (N4–H4A···N1, 2.85 Å and C3–H3···O3, 3.28 Å) discrete hydrogen bond interactions, stabilizing the 3D hydrogen bonding network. The neighboring layers are further held together via π–π interactions (∼4.2 Å) to generate a 3D structure (Figure 9b,c and Table S7).

These are the first examples of the oxicam salts with 4AP and 4DMP; the salts of LRM and TNM with PPZ are known.^31,32^ Comparing the crystal structures of the four oxicam-PPZ salts reveals that they all have a 2:1 stoichiometry. The PPZH_2_^2+^ dication lies over an inversion center, with a proton abstracted from each of the oxicam molecules, and it bridges the two oxicam anions through N^+^–H···O^–^ hydrogen bonds. Notably, for the MEL-PPZ salt, the PPZH_2_^2+^ dication is involved in more hydrogen bonding interactions than the other three oxicams.

### Physical Characterization

The PXRD patterns of the PRM system and the MEL system are shown in Figures S4 and S12, respectively. For all six salts, the experimental PXRD patterns match the theoretical patterns obtained from the SCXRD analysis, revealing that these salts were reproduced in bulk quantities by the slurry method.

FT-IR spectra of PRM and MEL systems are shown in Figures S3 and S11, respectively. The new crystalline solids exhibit different vibrational frequencies compared with those of the pure API and the salt formers, for example, for PRM, the characteristic absorption peak at 1628 cm^–1^ assigned to the C=O stretching vibration is red-shifted to 1626, 1618, and 1615 cm^–1^ in PRM-4AP, PRM-4DMP, and PRM-PPZ salts, respectively. For MEL, the C=O stretching vibration is red-shifted from 1617 to 1614 (MEL-4AP), 1611 (MEL-4DMP), and 1616 cm^–1^ (MEL-PPZ). Furthermore, the unencumbered −NH stretch in PRM (3337 cm^–1^) and MEL (3287 cm^–1^) is lost in the PRM or MEL salts, revealing that the −NH group is engaged in the formation of hydrogen bonds. These changes suggest the reconstruction of hydrogen bond networks in those solids and indicate the formation of new crystalline solids.

DSC and TGA studies were conducted on the six salts and their individual components to obtain the melting points and decomposition temperatures. The melting trace and decomposition behavior of each salt and the starting materials are given in the Supporting Information (Figures S1 and S2 for the PRM system and Figures S9 and S10 for the MEL system). Each salt shows a single sharp endothermic peak, suggesting that each product is in a homogeneous phase. Moreover, the TGA traces indicate that no solvent or water molecule is involved in the crystal lattice of these salts.

### Solubility Studies

**Figure 10:**
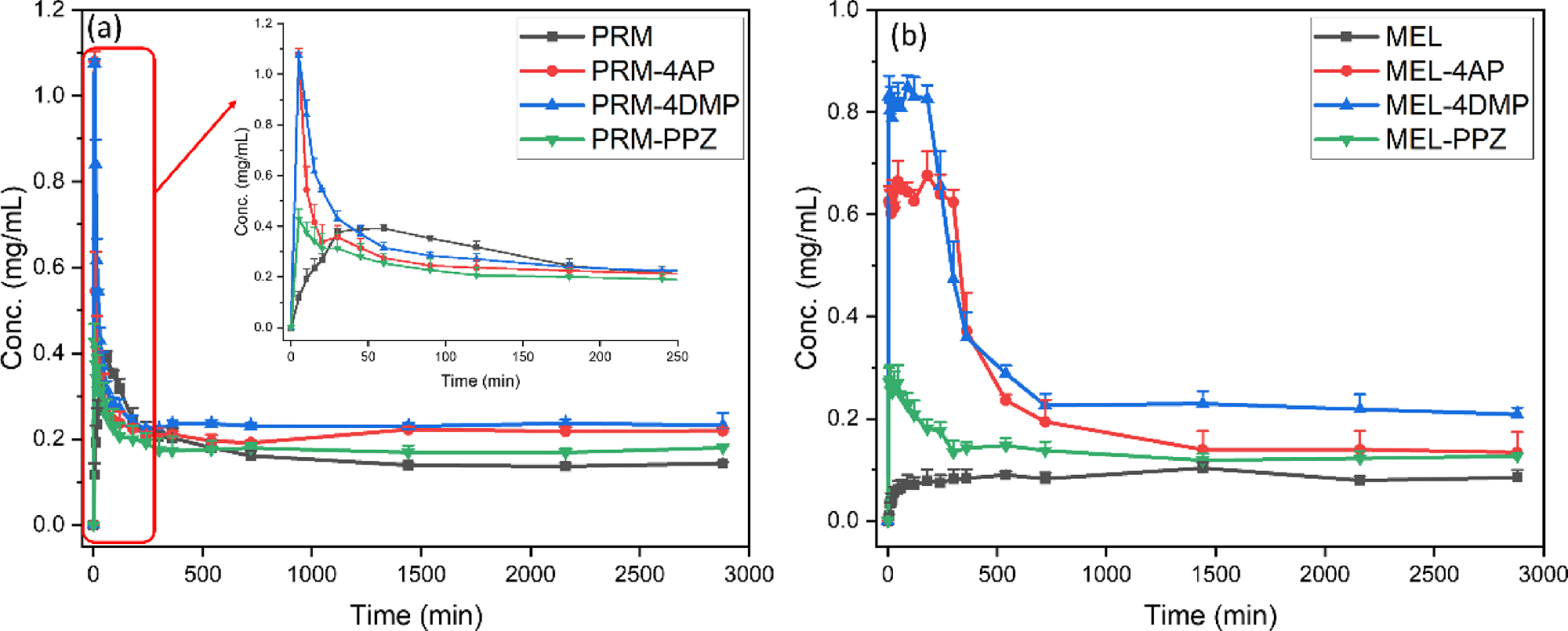
Solubility profiles of (a) PRM, PRM-4AP, PRM-4DMP, and PRM-PPZ and (b) MEL, MEL-4AP, MEL-4DMP, and MEL-PPZ.

PRM is dissolved and absorbed mainly in the intestine (in pH 6–8), and MEL undergoes significant degradation at lower pH (<3).^21,82^ Therefore, solubility tests of PRM, MEL, and their salts were performed in sodium phosphate buffer solutions (pH = 6.5) to investigate the ability of salt formation to improve the solubility of the poorly water-soluble APIs. As shown in Figure 10a, pure PRM reaches its highest solubility (0.39 mg mL^–1^) at 60 min. Subsequently, the concentration of pure PRM decreases slowly over time due to the crystal transformation from PRM anhydrate to PRM monohydrate and forms a plateau (0.14 mg mL^–1^). Similar behavior is observed for theophylline and caffeine.^83^ All the PRM salts exhibit the “spring and parachute” phenomenon,^84^ in that they dissolve faster than pure PRM and reach their maximum solubility within 5 min, and then the solubility decreases slowly over time owing to the transformation of the salts into the less soluble PRM monohydrate in solution (see Figure S7 for PXRD analysis confirming formation of the monohydrate). The dissolved PRMs of PRM-4AP, PRM-4DMP, and PRM-PPZ salts are 1.08, 1.07, and 0.43 mg mL^–1^, which are 2.8, 2.8, and 1.1 times higher than that of the anhydrous PRM, respectively (Table 4). Looking at Table 1, while some reported cocrystals and salts do not show any solubility advantage, these results are similar to the majority of the PRM salts and cocrystals in the literature, demonstrating enhanced solubility performance.

**Table 4:** Melting Point (T_m_) and Dissolved API of PRM, MEL, and the Six Salts

solids	*T*_m_ (°C)	dissolved PRM (mg mL^–1^)	solids	*T*_m_ (°C)	dissolved MEL (mg mL^–1^)
PRM	198–200	0.39 ± 0.01	MEL	259 (dec)	0.08 ± 0.02
PRM-4AP	213–216	1.08 ± 0.02	MEL-4AP	227–232	0.68 ± 0.05
PRM-4DMP	207–209	1.07 ± 0.01	MEL-4DMP	212–216	0.85 ± 0.02
PRM-PPZ	208–213	0.43 ± 0.04	MEL-PPZ	246–251	0.28 ± 0.03

Similarly, the solubility and dissolution rate of MEL are significantly enhanced by salt formation. As shown in Figure 10b, pure MEL dissolves slowly and reaches equilibrium (0.08 mg mL^–1^) at 90 min. The “spring and parachute” phenomenon is also observed for all the MEL salts, with PXRD analysis of the solid residues collected after the solubility experiments indicating that these undissolved solids had transformed to MEL (Figure S15). The time to maximum dissolved concentration of the salts is extended to 5 min, demonstrating the remarkably improved dissolution rate in comparison with that of pure MEL. The dissolved MEL of MEL-4AP, MEL-4DMP, and MEL-PPZ salts is 8.1, 10.2, and 3.3 times higher than that of the pure MEL, respectively. Furthermore, MEL-4DMP and MEL-4AP can maintain the supersaturation state for more than 200 and 300 min, respectively, indicating that the two salts of MEL could be promising formulations for achieving extended release without using polymers.^85,86^ These two salts have better solubility behavior when compared to most reported MEL cocrystals and salts, although the MEL aspirin cocrystal has significantly improved solubility compared to all other systems (Table 1).^48^

More recently, there have been reports of a correlation between the melting point and the solubility of cocrystals.^87,88^ In this study, no correlation was identified when examining the melting point and solubility of PRM salts; however, a semiempirical negative correlation between the drug melting point and drug solubility was found in the MEL system (Table 4).^62,88^ The melting point of the MEL salts increase in the following order: MEL-4DMP < MEL-4AP < MEL-PPZ, while the apparent solubility increases in the opposite order: MEL-PPZ < MEL-4AP < MEL-4DMP.

### Luminescence Studies

**Figure 11:**
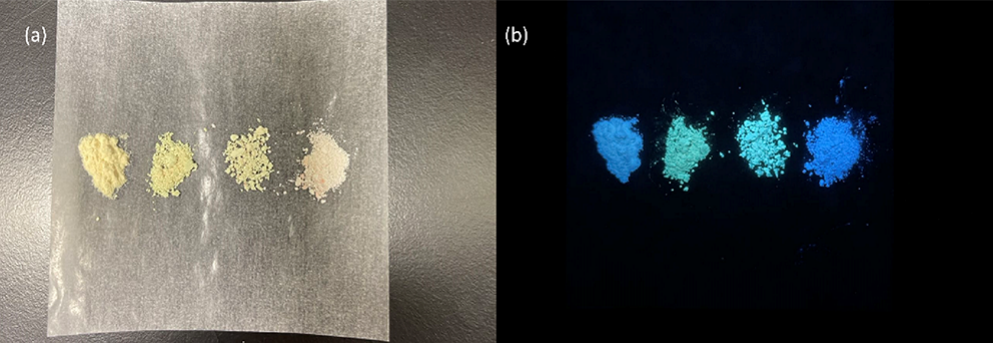
Photographs of PRM solids (from left to right: PRM, PRM-4AP, PRM-4DMP, and PRM-PPZ): (a) under white light illumination and (b) under a UV (365 nm) lamp.

**Figure 12:**
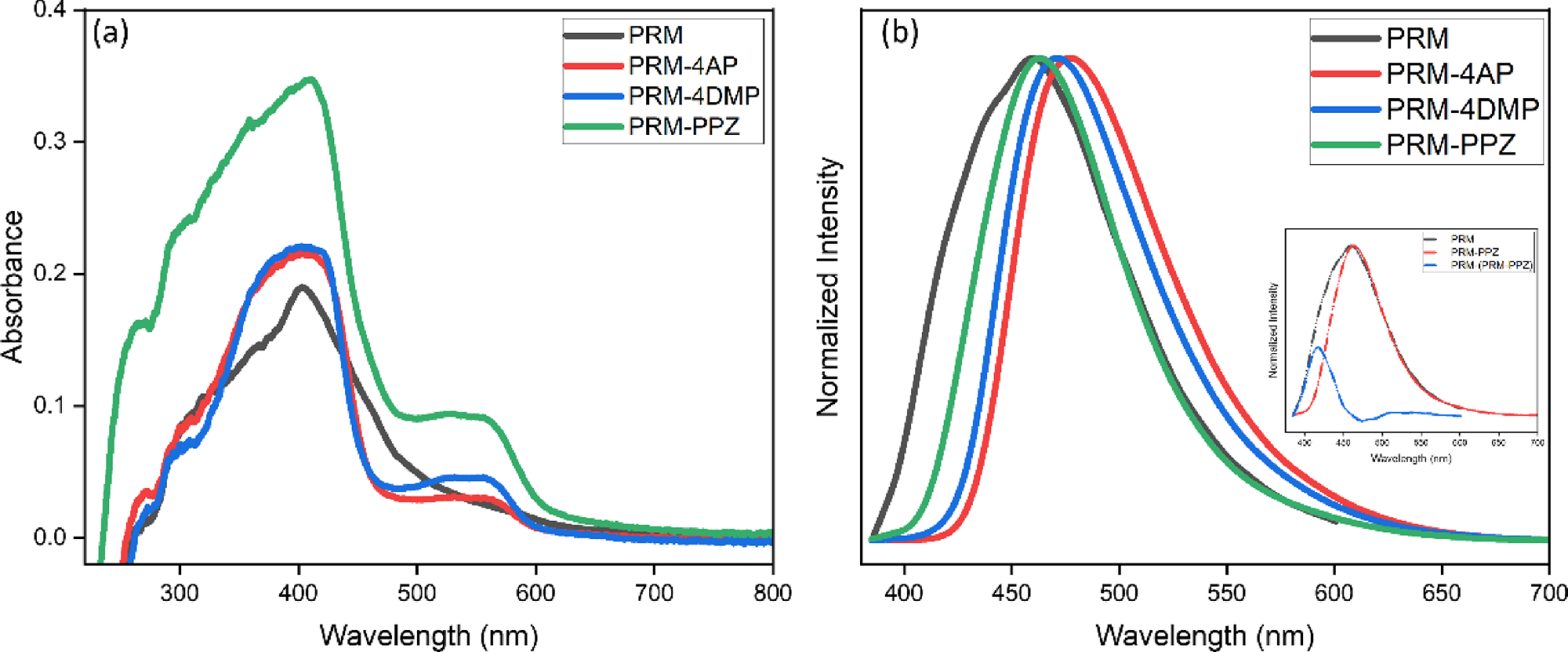
(a) Solid-state UV–vis absorption spectra and (b) normalized fluorescence spectra (excited at 365 nm) of PRM, PRM-4AP, PRM-4DMP, and PRM-PPZ. The blue line in the inset is the difference between the normalized fluorescence spectrum of PRM and that of PRM-PPZ, showing an apparent emission peak at about 417 nm in the PRM fluorescence.

It is known that PRM is fluorescent in dilute solution,^59^ and we observed that PRM and the three salts exhibit relatively strong solid-state luminescence. As shown in Figure 11, PRM, PRM-4AP, and PRM-4DMP are yellow and PRM-PPZ is pale pink in color under white light illumination. However, upon irradiating with UV light, the PRM-4AP and PRM-4DMP salts exhibit strong cyan fluorescence, while PRM and PRM-PPZ show blue fluorescence. Solid-state UV–vis absorption spectra for PRM and its three salts were measured to further investigate the luminescent properties (Figure 12a). The wavelength of maximum absorption for all four solids is ca. 405 nm, and there is weaker absorption which peaks around 560 nm in all three salts. In addition, the absorption bands for PRM-4AP and PRM-4DMP demonstrate a broad trend with a slight red shift in the higher energy absorption band. Based on analysis by Lu et al. on a different luminescent system,^63^ this suggests that the charge transfer interaction in these two salts is stronger than that in the PRM-PPZ salt. Solid-state fluorescence spectra and quantum yields of the four PRM solids are shown in Table 5 and Figure 12b. Both PRM-4AP and PRM-4DMP salts display significantly red-shifted spectra and higher quantum yields compared to those of PRM, while a slight red shift and lower quantum yield are observed for PRM-PPZ. The difference of the luminescent properties among the three salts could be attributed to different fluorescence mechanisms, such as aggregate quenching,^89^ or greater competition from nonradiative relaxation processes in the case of PRM-PPZ.

**Table 5:** Comparison of the Maximum Fluorescence Emission Wavelengths, Fluorescence Quantum Yields, and Contributions of π–π and Hydrogen Bonding of PRM, PRM-4AP, PRM-4DMP, and PRM-PPZ

	λ_em_^max^ (nm)	ΦF^a^	π–π^b^(%)	hydrogen bonding^b^(%)
PRM	460	0.614	6.2	33.8
PRM-4AP	477	0.685	5.1	33.9
PRM-4DMP	471	0.660	2.2	33.7
PRM-PPZ	463	0.408	2.3	32.7

a Fluorescence quantum yields excited at 365 nm.

b Refers to the contribution of π–π interactions and hydrogen bonding in the crystal structures. Values are those obtained from Hirshfeld surface calculations (Table S4).

From the structural perspective, PRM demonstrates different conformations and intramolecular interactions before and after forming salts (Figure 1). In pure PRM, the hydrogen atom from the hydroxyl group forms an intramolecular hydrogen bond with the oxygen atom from the carbonyl group. In the deprotonated PRM, there is an intramolecular hydrogen bond between the hydrogen atom from secondary amine and the oxygen ion. Based on the excited-state intramolecular proton transfer (ESIPT) theory and internal charge transfer theory,^64,90,91^ we suggest that the fluorescence mechanism for PRM salts could be proton-transfer-induced enhanced luminescence with a moderate Stokes shift. Support for an ESIPT mechanism is seen in the apparent short wavelength emission peak in PRM that occurs on top of the longer wavelength emission, seen in PRM and its salts (Figure 12). Here, the short wavelength peak in PRM would match an enol tautomer, while the longer wavelength emission in PRM and its salts would correspond to the lower-energy keto state. After proton transfer, the new conformation and new intramolecular interactions lead to the red-shifted spectra of PRM-4AP and PRM-4DMP, as well as the higher quantum yields compared with those of pure PRM. In addition, the maximum emission wavelength of PRM-4DMP is slightly blue-shifted in comparison with that of PRM-4AP, which could be attributed to the relatively weaker π–π interactions.^62,92^ The stronger intermolecular interactions suppress vibrational relaxation to enhance the quantum yields;^93^ consequently, PRM-4AP presents a higher quantum yield compared with that of PRM-4DMP. However, the difference in the emission wavelength maxima between PRM and PRM-PPZ is not as significant as the difference between PRM and PRM-4AP or PRM-4DMP, which suggests that the fluorescence performance of PRM-PPZ could also be affected by other factors, such as the electron distribution in the ground and excited states.

**Figure 13:**
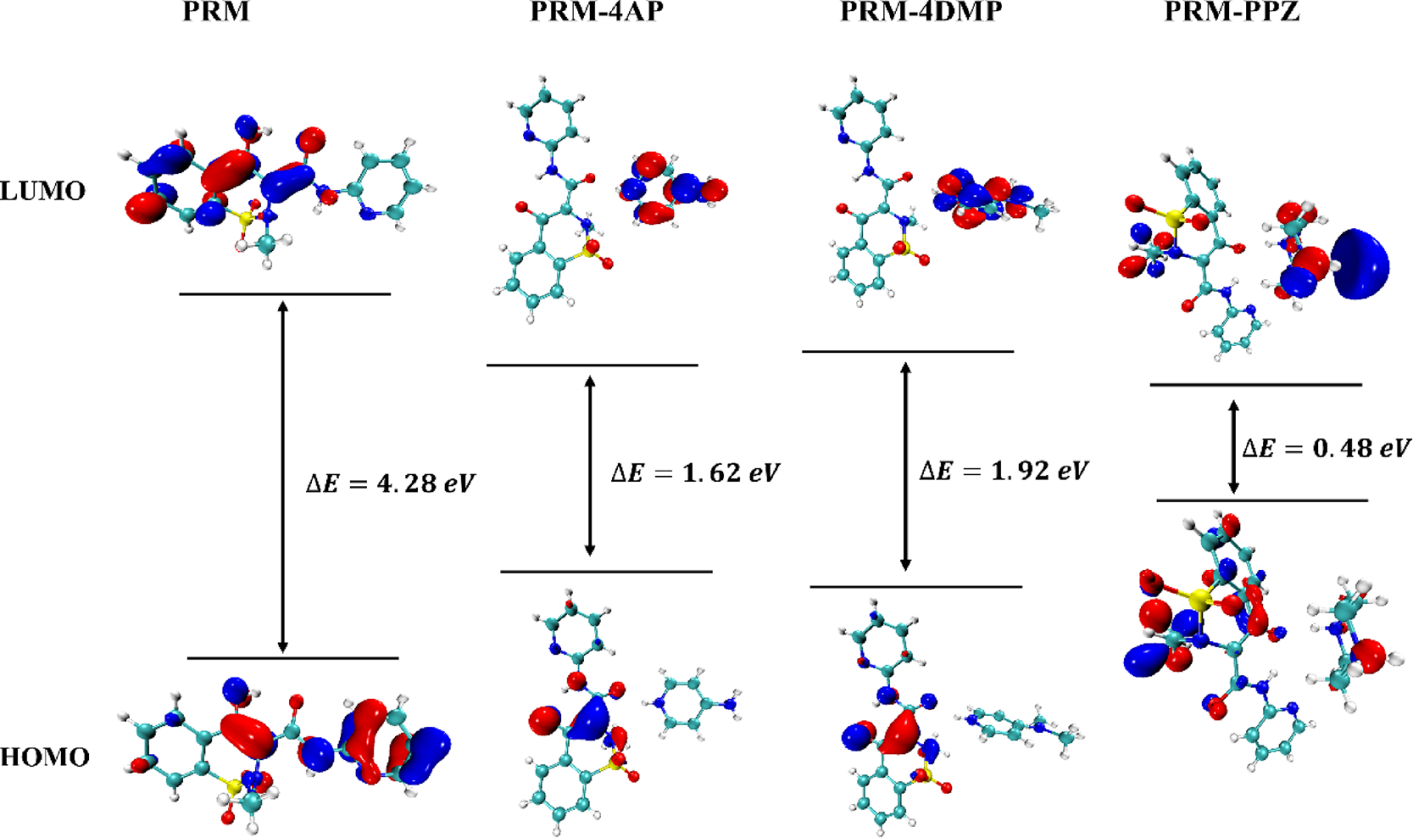
Molecular orbital plots of the HOMOs and LUMOs of PRM, PRM-4AP, PRM-4DMP, and PRM-PPZ.

FMOs have been used to explain the reactivity in chemical systems and to predict the most reactive position in conjugated systems.^94−96^ A comparison of the FMOs has been undertaken to see if it provides an explanation for the luminescent properties of PRM and these three salts. As shown in Figure 13, the highest occupied molecular orbital (HOMO) of PRM is located over the skeleton of the PRM molecule, except for the phenyl ring. In contrast, it is the pyridyl ring that is not involved in the lowest unoccupied molecular orbital (LUMO). For PRM-4AP and PRM-4DMP, the HOMOs are mainly restricted in the middle of PRM, especially around two oxygens and the chemical bonds in between, suggesting that these are the most reactive positions. The LUMOs are associated with the cations 4APH^+^ and 4DMPH^+^, respectively. Therefore, the deprotonation of PRM plays an important role in the structural, electronic, and luminescent changes of the PRM-4AP and PRM-4DMP systems, which also supports our proposal that the mechanism for the luminescent performance of PRM-4AP and PRM-4DMP is credited to the proton transfer of PRM. In addition, the larger energy gap of PRM-4DMP also contributes to the blue-shifted spectrum in comparison to PRM-4AP.^97^

However, in the PRM-PPZ salt, although PRM is deprotonated and presents a similar conformation to the previous two salts, the distribution of the HOMO and LUMO varies significantly. Both are mainly located around the methyl group, the benzothiazine moiety of PRM, and the PPZH_2_^2+^ dication, suggesting that the proton transfer sites are not the most reactive positions in this conjugated system. Therefore, the observed luminescence is likely reduced by some factors, such as crystal packing and molecular arrangement, that are known to quench fluorescence in the solid state.

MEL can exhibit fluorescence in the solution state;^98^ however, it is weakly fluorescent (Φ_F_ = 0.14, Figure S16) in the crystal state, possibly indicative of fluorescence quenching caused by aggregation (ACQ).^64^ The MEL-4AP, MEL-4DMP, and MEL-PPZ salts present almost no fluorescence (Φ_F_ = 0.008, 0.044, and 0.016, respectively), again indicating quenching due to aggregation.

## Conclusions

In summary, six new pharmaceutical salts of PRM and MEL with three organic counterions (4AP, 4DMP, and PPZ) were successfully synthesized and characterized by various solid-state analytical techniques, including SCXRD, PXRD, DSC, TGA, and IR. In the solubility tests, the apparent solubility of all six salts was enhanced relative to that of the parent molecule (PRM/MEL), and the dissolution rate of all of six salts was also improved significantly. The salts exhibit similar solid-state luminescent properties to those of PRM and MEL. The proton-transfer-induced enhanced luminescence with a large red shift could be used to explain the luminescence mechanism of PRM-4AP and PRM-4DMP. For PRM-PPZ, the mechanism could be the combination of the proton transfer process with some quenching process. Hirshfeld surface analysis and HOMO–LUMO analysis were also employed to further investigate the different luminescent behaviors of PRM solids. Overall, this study revealed that salt formation by using organic counterions is an effective approach to improve the solubility behavior of poorly water-soluble APIs. Furthermore, the luminescent properties of organic fluorophores can be altered and modified by forming salts involving proton transfer. In this example, exposure of samples under UV illumination provides a convenient and useful tool to examine the synthesis of new crystalline materials.

## Supporting Information Available

DSC traces; TGA traces; IR spectra; PXRD data; ellipsoid plots; angle formed between the methyl hydrogen and the plane of a sp^2^ oxygen in PRM and MEL salts; crystallographic parameters of PRM and MEL salts; PXRD patterns of residual solids after solubility experiments; 3D d_norm_ surfaces and 2D fingerprint plots of PRM and MEL in the salts; photographs of MEL solids under daylight and a UV lamp; molecular orbital plots of the HOMOs and LUMOs of MEL and its salts; and summary of the various contact contributions to the PRM and MEL Hirshfeld surface area in pure PRM, MEL, and salts (PDF)
