# Supplementary material for: Pharmaceutical Salts of Piroxicam and Meloxicam with Organic Counterions
Source: Cryst Growth Des. 2022 Oct 21;22(11):6504–20. doi: 10.1021/acs.cgd.2c00722 (PMC9933440; doi:10.1021/acs.cgd.2c00722)
Supplement: Supplementary file 1 — cg2c00722_si_001.pdf [file cg2c00722_si_001.pdf]

Supporting Information

Pharmaceutical Salts of Piroxicam and Meloxicam with Organic Counterions

Shan Huang,[†] *Dean S. Venables,*[‡] *and Simon E. Lawrence* *[,†]

[†]School of Chemistry, Analytical and Biological Chemistry Research Facility, Synthesis and Solid State Pharmaceutical Centre, University College Cork, Cork, T12 K8AF, Ireland

[‡]School of Chemistry and Environmental Research Institute, University College Cork, Cork, T12 K8AF, Ireland

*E-mail: simon.lawrence@ucc.ie

Content

Analytical data for the PRM salts	2
Analytical data for the MEL salts	12

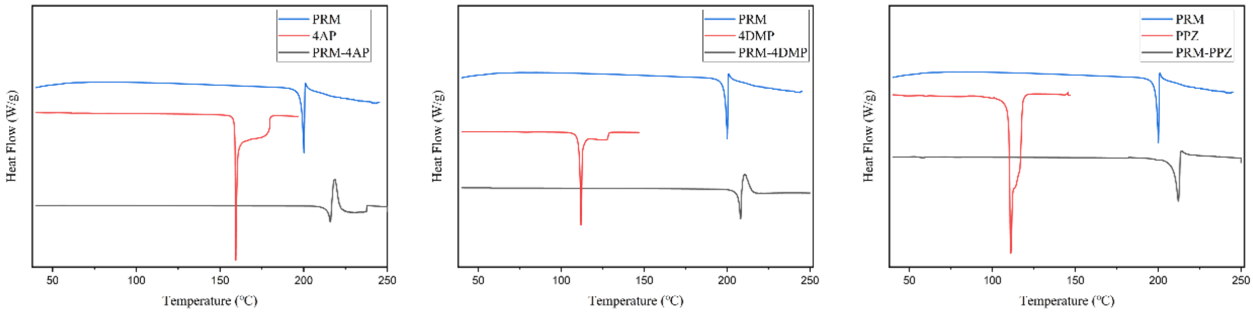

Figure S1. DSC traces of PRM (blue), salt formers (4AP, 4DMP and PPZ; red) and salts (PRM-4AP, PRM-4DMP and PRM-PPZ; black).

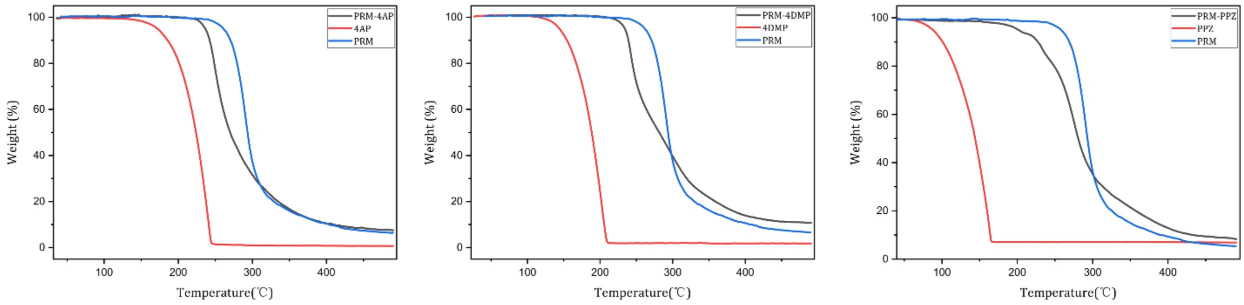

Figure S2. TGA traces of PRM (blue), salt formers (4AP, 4DMP and PPZ; red) and salts (PRM-4AP, PRM-4DMP and PRM-PPZ; black).

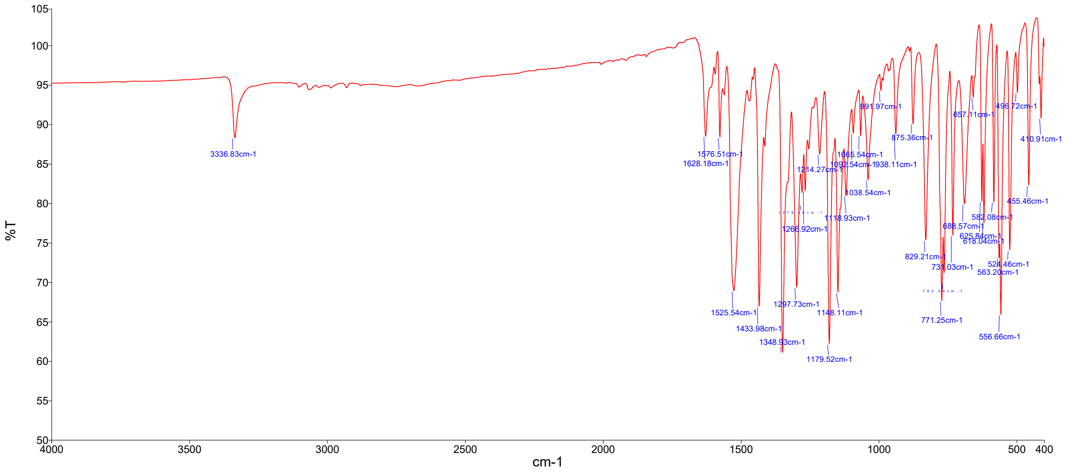

(a)

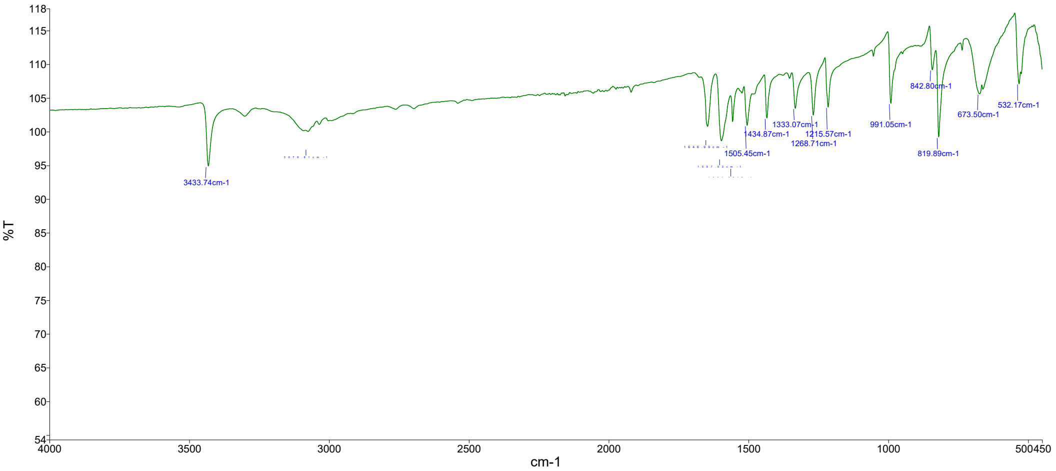
%T
cm-1
3433.74cm-1
1505.45cm-1
1434.87cm-1
1333.07cm-1
1268.71cm-1
1215.57cm-1
991.05cm-1
842.80cm-1
819.89cm-1
673.50cm-1
532.17cm-1

(b)

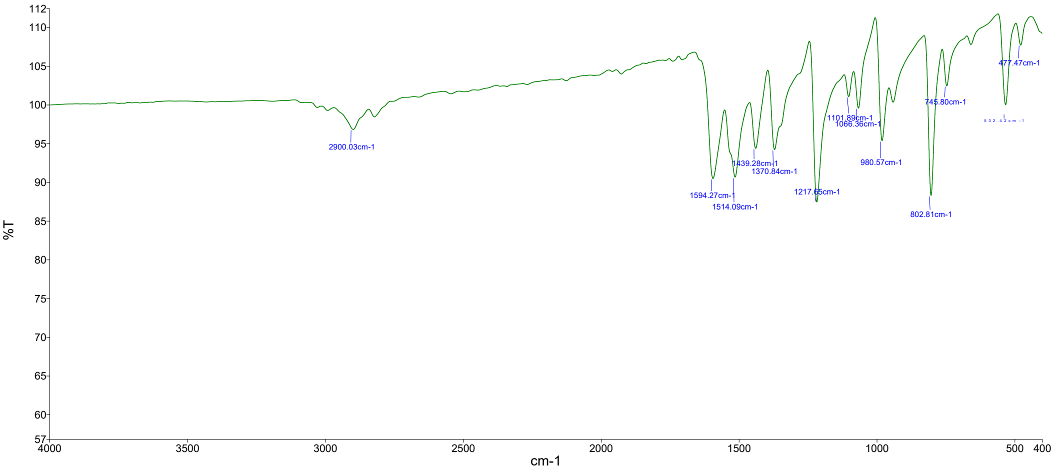
%T
cm-1
2900.03cm-1
1594.27cm-1
1514.09cm-1
1439.28cm-1
1370.84cm-1
1217.65cm-1
1101.89cm-1
1066.36cm-1
980.57cm-1
802.81cm-1
745.80cm-1
477.47cm-1

(c)

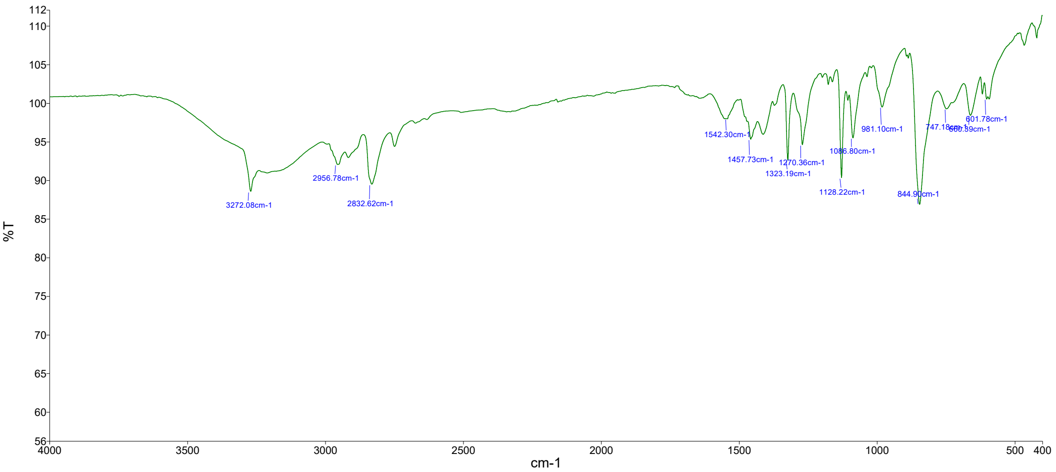
%T
cm-1
3272.08cm-1
2956.78cm-1
2832.62cm-1
1542.30cm-1
1457.73cm-1
1323.19cm-1
1270.36cm-1
1128.22cm-1
1086.80cm-1
981.10cm-1
844.90cm-1
747.18cm-1
601.78cm-1

(d)

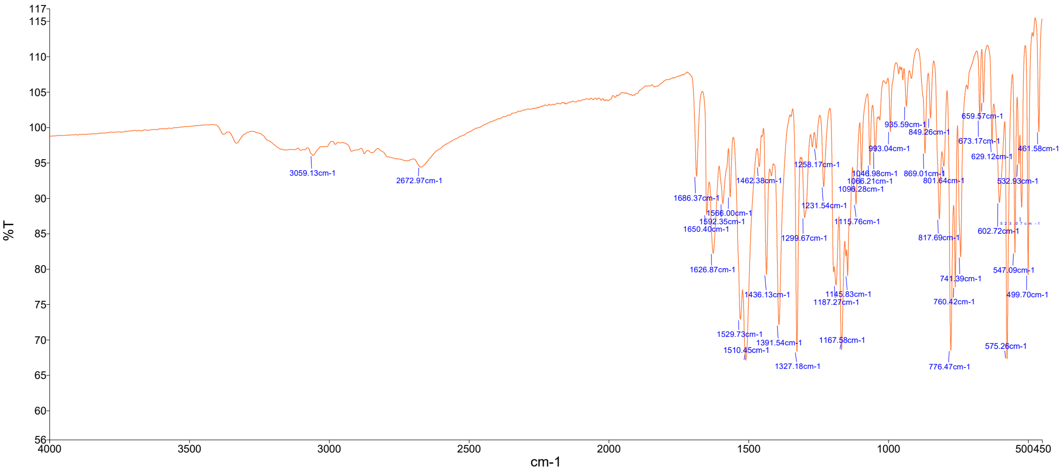

(e)

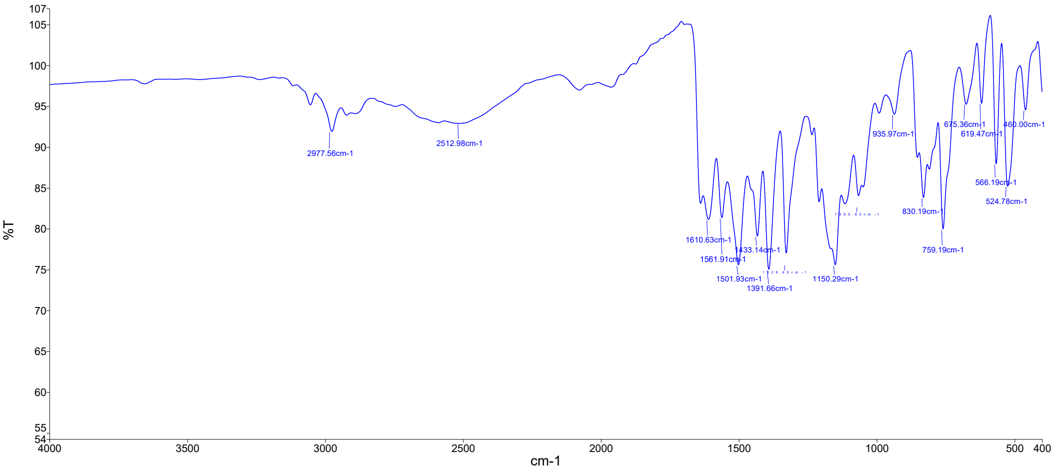

(f)

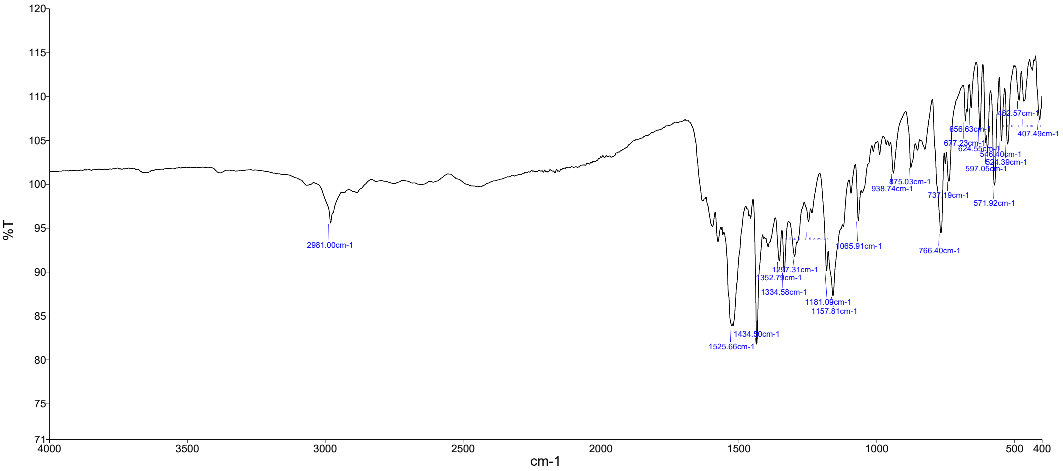

(g)

Figure S3. IR spectra of (a) PRM, (b) 4AP, (c) 4DMP, (d) PPZ, (e) PRM-4AP, (f) PRM-4DMP and (g) PRM-PPZ.

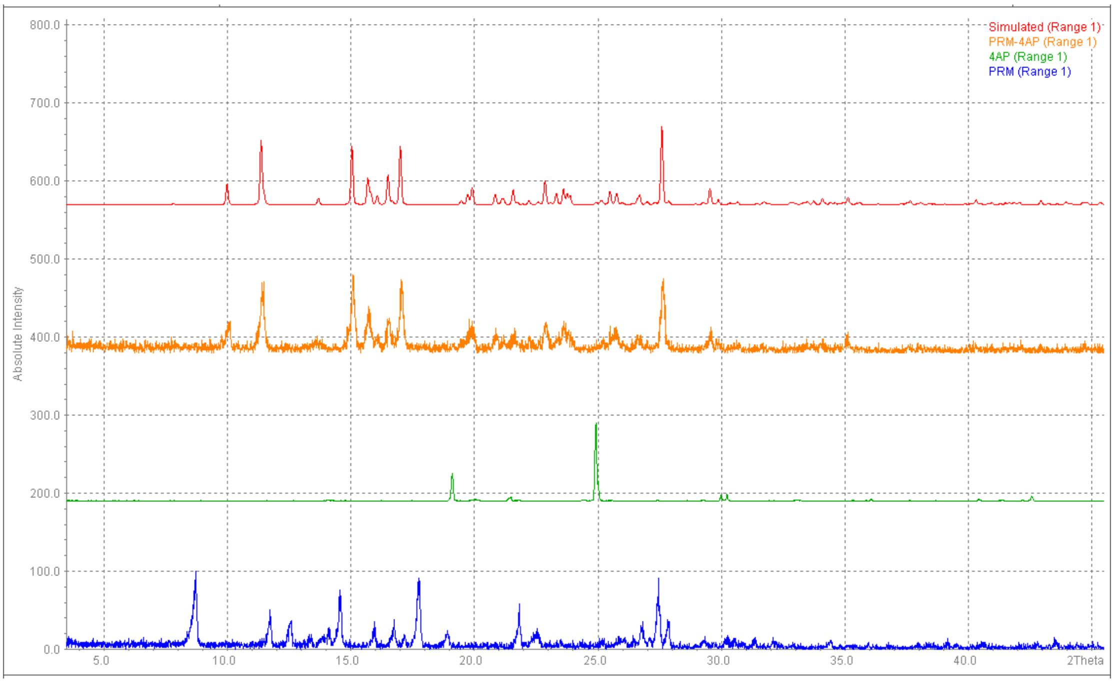
Simulated (Range 1)
PRM-4AP (Range 1)
4AP (Range 1)
PRM (Range 1)
Absolute Intensity
800.0
700.0
600.0
500.0
400.0
300.0
200.0
100.0
0.0
5.0
10.0
15.0
20.0
25.0
30.0
35.0
40.0
2Theta

(a)

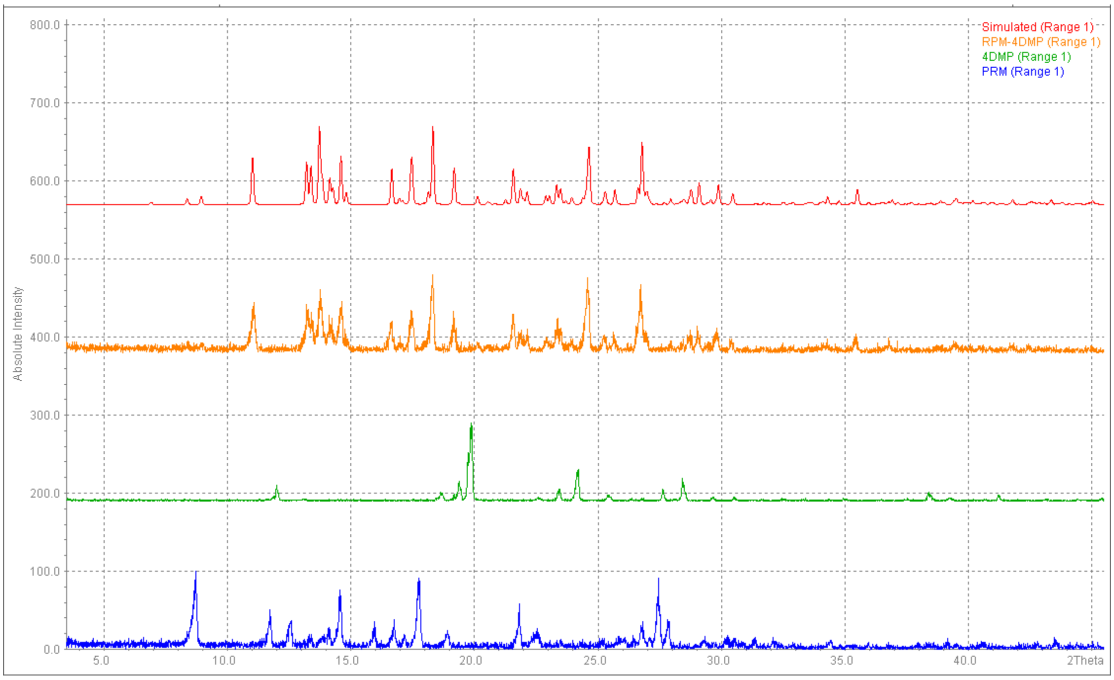
Simulated (Range 1)
RPM-4DMP (Range 1)
4DMP (Range 1)
PRM (Range 1)
Absolute Intensity
800.0
700.0
600.0
500.0
400.0
300.0
200.0
100.0
0.0
5.0
10.0
15.0
20.0
25.0
30.0
35.0
40.0
2Theta

(b)

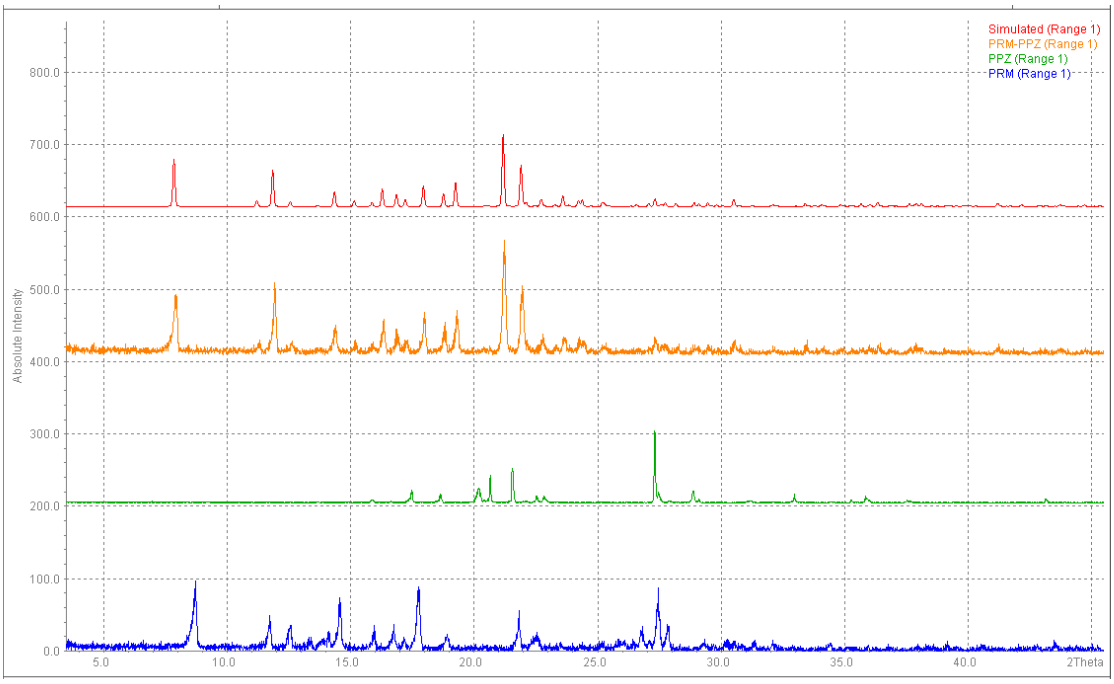

(c)

Figure S4. (a) PXRD patterns of PRM (blue), 4AP (green), PRM-4AP salt (orange) and simulated pattern from the crystal structure (red), (b) PXRD patterns of PRM (blue), 4DMP (green), PRM-4DMP salt (orange) and simulated pattern from the crystal structure (red) and (c) PXRD patterns of PRM (blue), PPZ (green), PRM-PPZ salt (orange) and simulated pattern from the crystal structure (red).

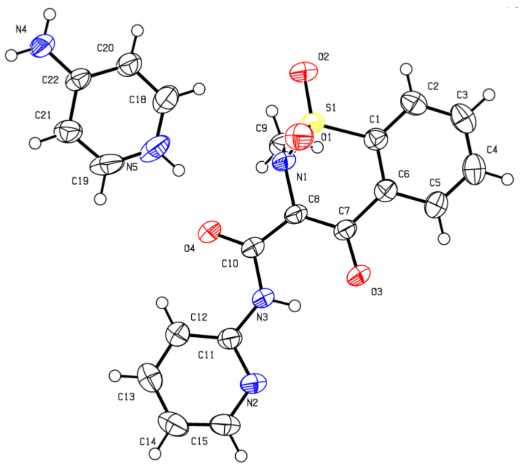

(a)

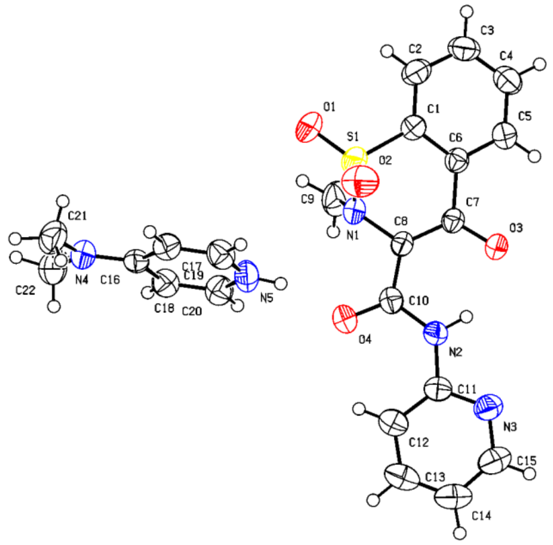

(b)

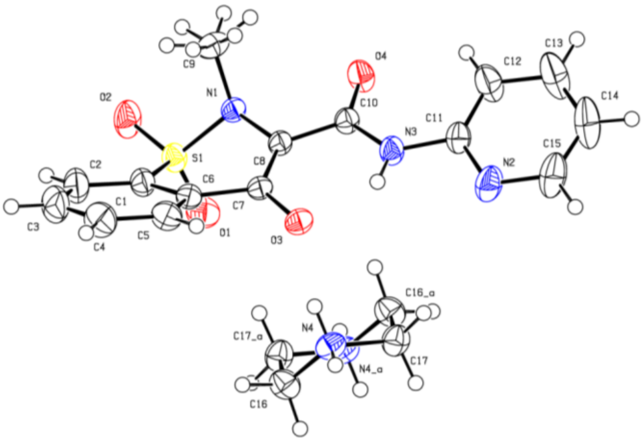

(c)

Figure S5. Ellipsoid plot of (a) PRM-4AP, (b) PRM-4DMP and (c) PRM-PPZ.

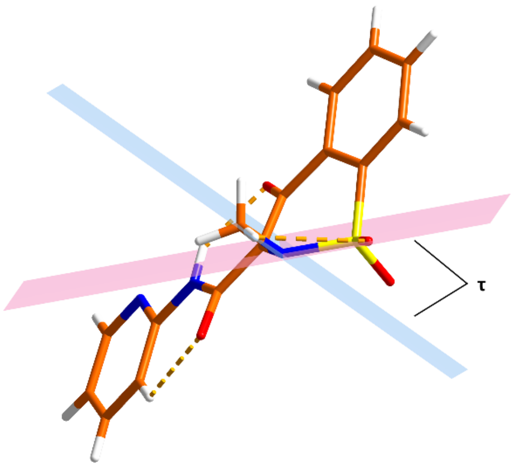

Solid	τ (°)
PRM-4AP	45.4
PRM-4DMP	45.6
PRM-PPZ	42.9

Figure S6. The angle (τ) formed between the methyl hydrogen and the plane of a sp^2 oxygen in PRM^- anion in PRM-4AP, PRM-4DMP and PRM-PPZ.

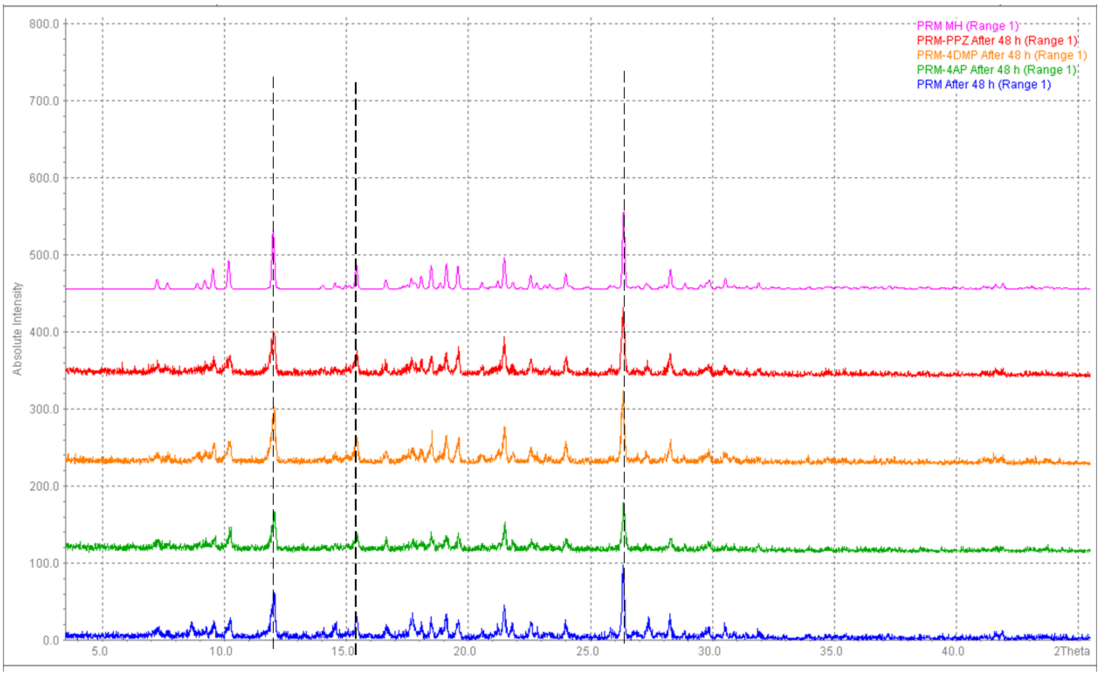

Figure S7. PXRD patterns of residual solids of PRM (blue), PRM-4AP (green), PRM-4DMP (orange), PRM-PPZ (red) after solubility experiments, and the simulated pattern from the crystal structure of PRM monohydrate (pink).

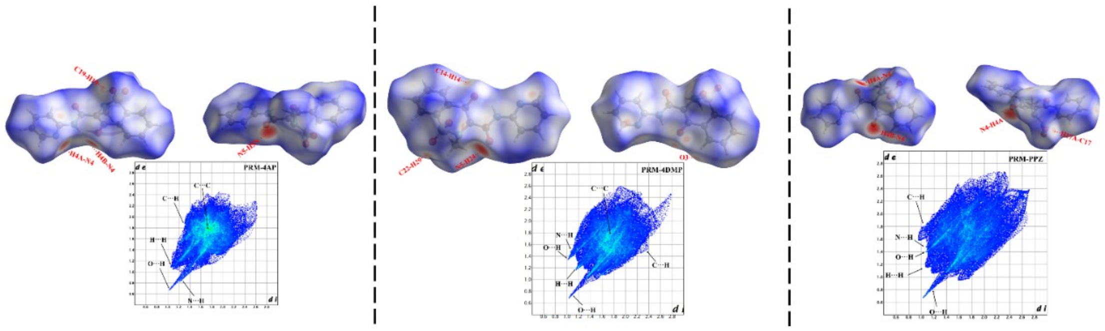

Figure S8. 3D d_{norm} surfaces and 2D fingerprint plots of PRM in PRM-4AP (left), PRM-4DMP (middle) and PRM-PPZ (right)

Table S1 Hydrogen bond and π–π interaction geometries (Å, °) in the PRM-4AP salt.

	Type	D-H···A	D-H	H···A	D···A	D-H···A	ARU (J)
1	Intra	N3-H3N···O3	0.86	1.93	2.647	139	
2		N4-H4A···N2	0.86	2.18	2.991	157	1556.01
3		N4-H4B···O3	0.86	2.09	2.889	153	1556.01
4		N5-H5N···O4	0.86	1.83	2.653	159	1555.01
5	Intra	C9-H9B···O2	0.96	2.47	2.847	103	
6	Intra	C12-H12···O4	0.93	2.29	2.867	120	
7		C19-H19···O1	0.93	2.52	3.277	139	2666.01

Cg(I)	Cg(J)	Cg-Cg	Interplanar distance	Dihedral Angle	Beta	ARU (J)
2	5	4.1818(19)	3.5149(9)	10.50(11)	22.3	2566.02
5	2	4.1727(19)	3.0843(9)	10.50(11)	33	2666.02
3	3	3.6422(16)	3.3605(7)	0.02(8)	22.7	2775.01

[a] Symmetry codes: [1556] = x, y, 1+z; [2566] = -x, 1-y, 1-z; [2666] = 1-x, 1-y, 1-z; [2775] = 2-x, 2-y, -z. Cg2 represents the centroid of N2, C11-C15; Cg3 represents the centroid of C1–C6; Cg5 represents the centroid of N5, C18–C22. Cg(I) = plane number I; Cg–Cg = distance between ring centroids (Ang.). Beta is the displacement angle between the ring normal of plane I and the centroid vector.

Table S2 Hydrogen bond and π–π interaction geometries (Å, °) in the PRM-4DMP salt.

	Type	D-H···A	D-H	H···A	D···A	D-H···A	ARU (J)
1	Intra	N2-H23···O3	0.875(9)	1.907(8)	2.6504(10)	141.8(8)	
2		N5-H24···O4	0.872(9)	1.853(9)	2.6881(11)	159.8(8)	[1555.01]
3		N5-H24···N1	0.872(9)	2.599(9)	3.1549(12)	122.6(7)	[1555.01]
4		C5-H5···O3	0.93	2.51	3.3852(12)	157	[7666.01]
5	Intra	C12-H12···O4	0.93	2.27	2.8577(12)	121	
6		C14-H14···O3	0.93	2.56	3.4297(13)	156	[6646.01]
7		C19-H19···O2	0.93	2.55	3.3241(12)	141	[2656.01]
8	Intra	C9-H25···O1	0.96	2.49	2.8560(14)	102	
9		C22-H29···O1	0.96	2.44	3.3961(14)	172	[7566.01]

Cg(I)	Cg(J)	Cg-Cg	Interplanar distance	Dihedral Angle	Beta	ARU (J)
2	5	4.0738(9)	3.4027(4)	1.82(4)	34.9	[2656.02]

[a] Symmetry codes [7666.] = 3/2-x,3/2-y,1-z; [6646.] = 3/2-x, -1/2+y,3/2-z; [2656.] = 1-x, y, 3/2-z; [7566.] = 1/2-x, 3/2-y,1-z. Cg2 represents the centroid of N3, C11-C15 and Cg5 represents the centroid of N5, C16–C20. Cg(I) = plane number I; Cg–Cg = distance between ring centroids (Ang.). Beta is the displacement angle between the ring normal of plane I and the centroid vector.

Table S3 Hydrogen bond and π–π interaction geometries (Å, °) in the PRM-PPZ salt.

	Type	D-H···A	D-H	H···A	D···A	D-H···A	ARU (J)
1	Intra	N3-H3N···O3	0.86	1.91	2.6447	143	
2		N4-H4A···O4	0.89	1.81	2.6451	156	1545.01
3		N4-H4B···O3	0.89	1.82	2.6959	168	1555.01
4	Intra	C9-H9A···O2	0.96	2.45	2.871	106	
6	Intra	C12-H12···O4	0.93	2.32	2.892	119	
7		C17-H17A···O1	0.97	2.59	3.481	153	3556.01

[a] Symmetry codes: [1545.] = x, -1+y ,z; [3556.]= -x, -y, 1-z.

Table S4 Summary of the various contact contributions to the PRM Hirshfeld surface area in pure PRM and its salts.

	O-H	H-H	C-H	C-O	C-C	N-H	O-N	N-C	N-N	H-S	O-O
**PRM**	27.4	37.3	14.9	4.4	6.2	6.4	0.8	0.1	1.1	0	1.4
**PRM-4AP**	28.1	42.2	15.5	1.2	5.1	5.8	0.2	1.7	0.1	0	0
**PRM-4DMP**	26.7	43.9	18	1.1	2.2	7	0.2	0.5	0.2	0	0.2
**PRM-PPZ**	24.3	39.2	21.1	2.8	2.3	8.4	0.2	0	0	0.1	1.5

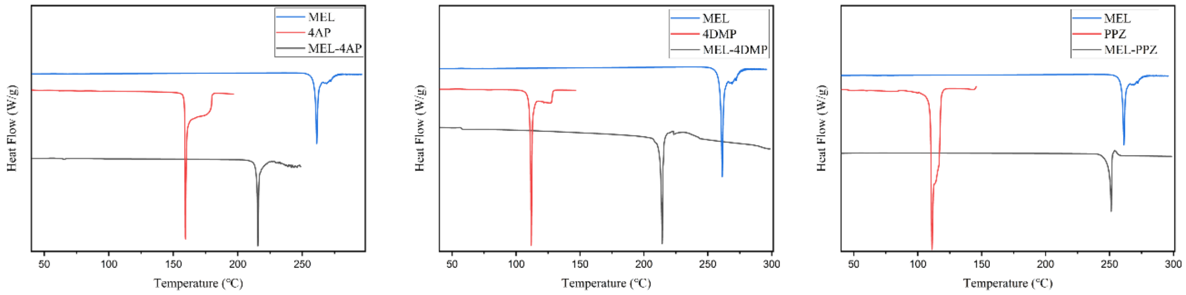

Figure. S9 DSC traces of MEL (blue), salt formers (4AP, 4DMP and PPZ; red) and salts (MEL-4AP, MEL-4DMP and MEL-PPZ; black).

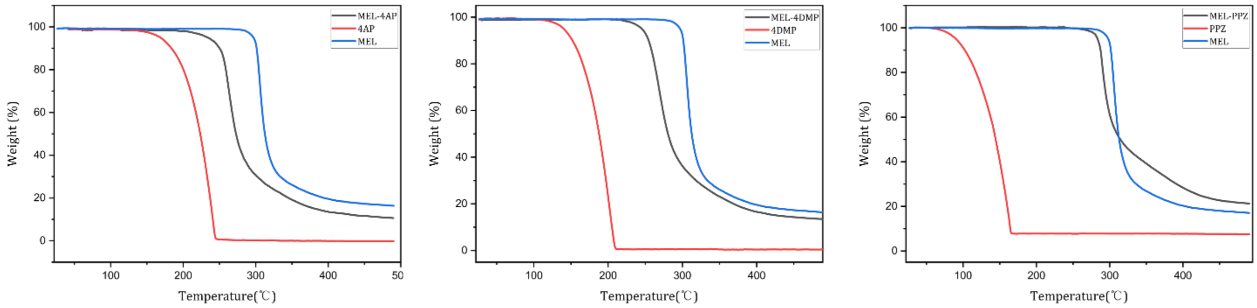

Figure. S10 TGA traces of MEL (blue), salt formers (4AP, 4DMP and PPZ; red) and salts (MEL-4AP, MEL-4DMP and MEL-PPZ; black).

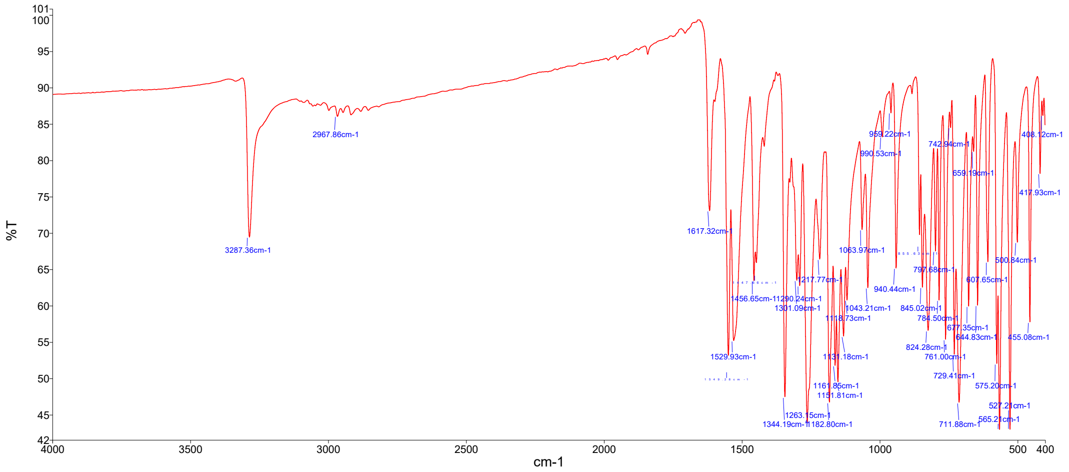

(a)

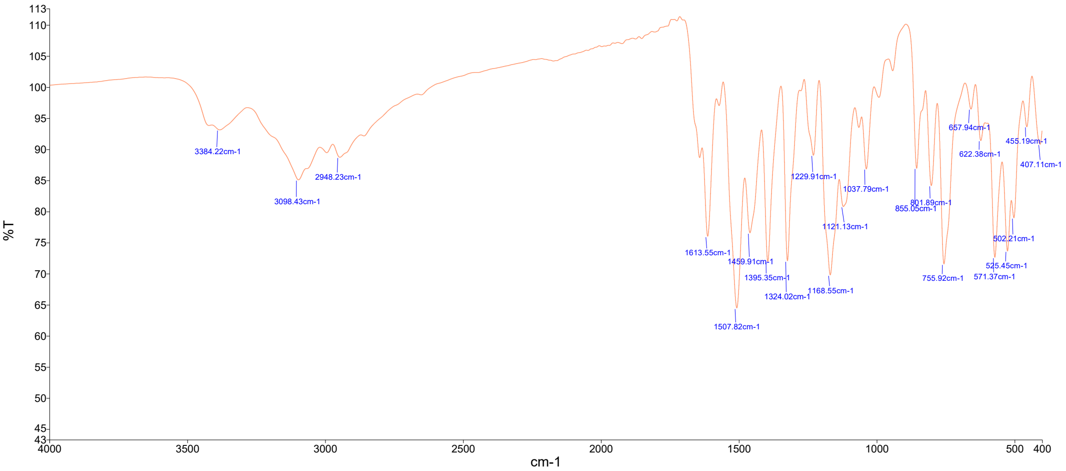

(b)

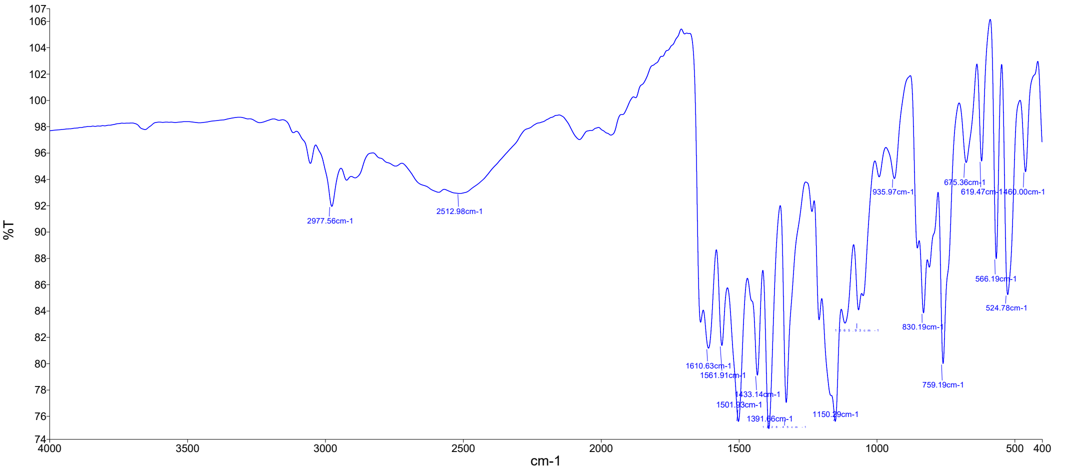

(c)

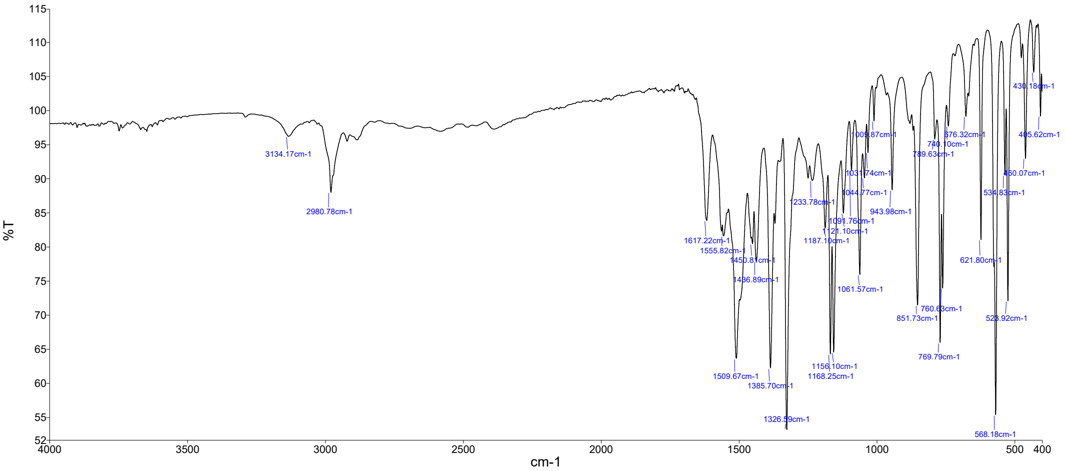

(d)

Figure S11. IR spectra of (a) MEL, (b) MEL-4AP, (c) MEL-4DMP and (d) MEL-PPZ.

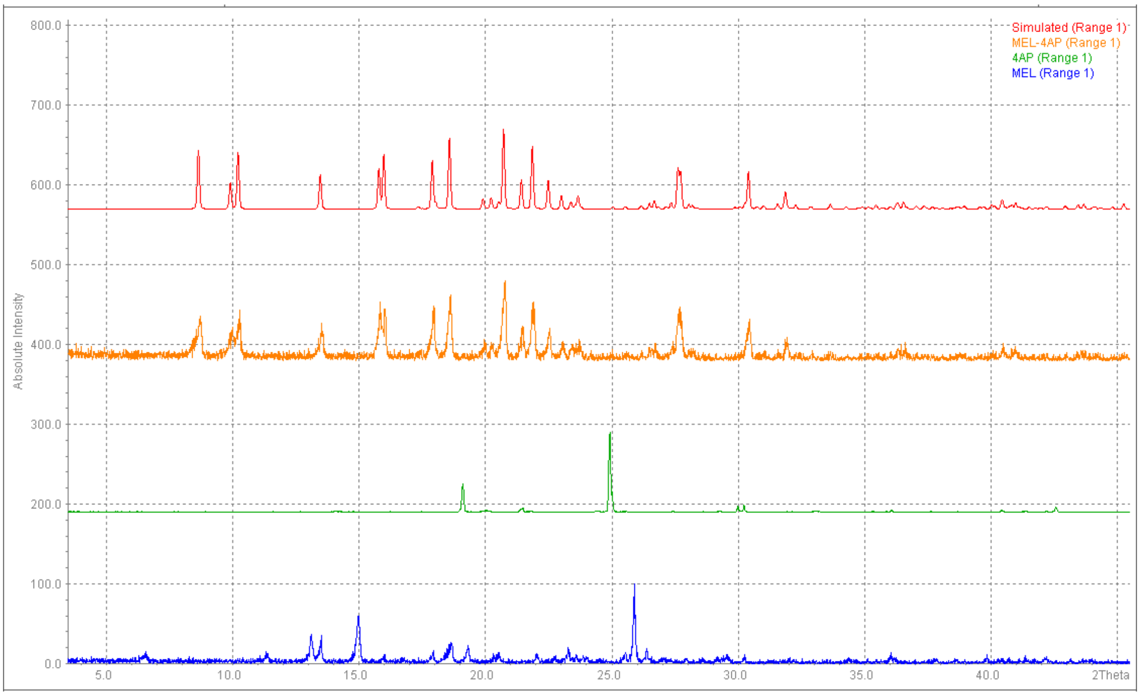
Simulated (Range 1)
MEL-4AP (Range 1)
4AP (Range 1)
MEL (Range 1)
Absolute Intensity
800.0
700.0
600.0
500.0
400.0
300.0
200.0
100.0
0.0
5.0
10.0
15.0
20.0
25.0
30.0
35.0
40.0
2Theta

(a)

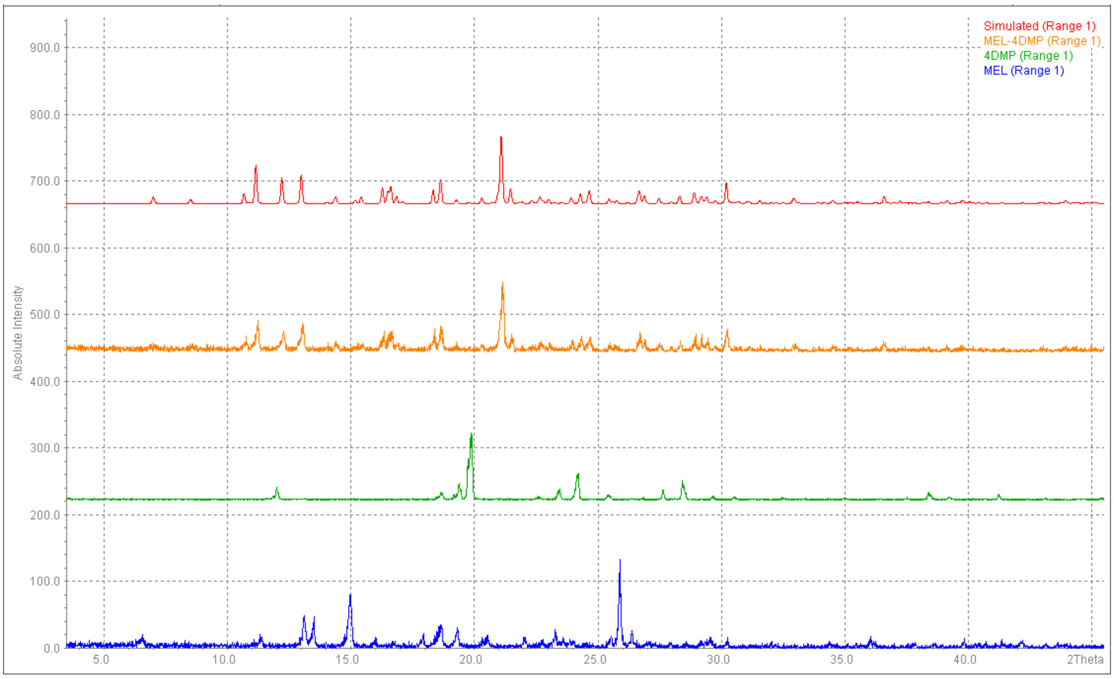
Simulated (Range 1)
MEL-4DMP (Range 1)
4DMP (Range 1)
MEL (Range 1)
Absolute Intensity
900.0
800.0
700.0
600.0
500.0
400.0
300.0
200.0
100.0
0.0
5.0
10.0
15.0
20.0
25.0
30.0
35.0
40.0
2Theta

(b)

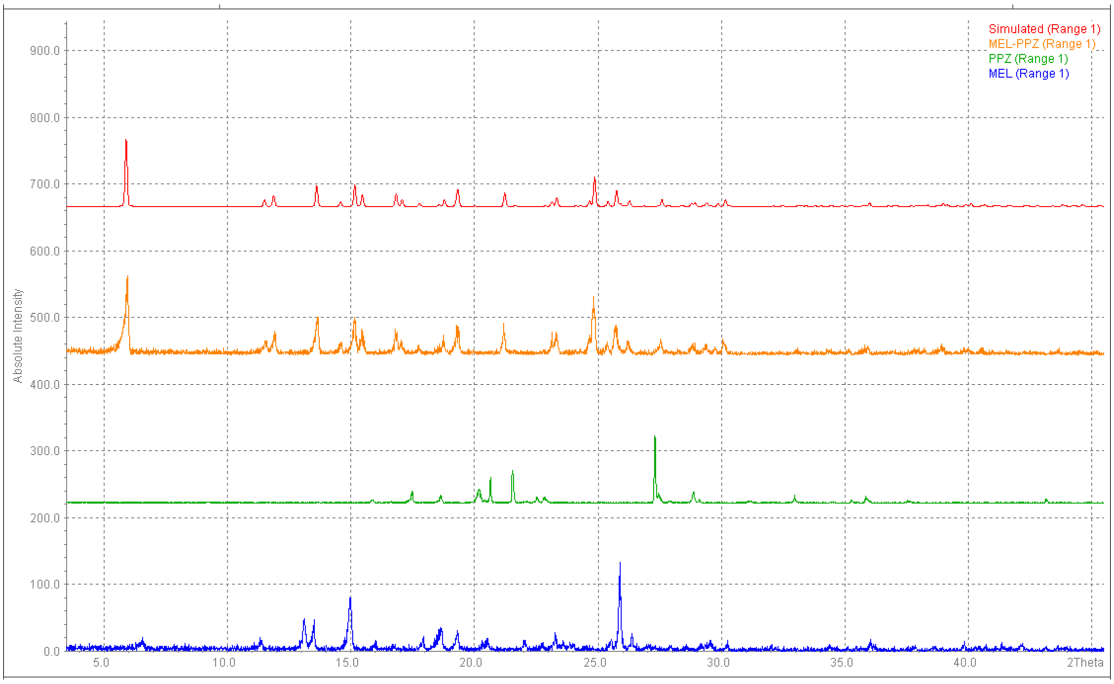

(c)

Figure S12. (a) PXRD patterns of MEL (blue), 4AP (green), MEL-4AP salt (orange) and simulated pattern from the crystal structure (red), (b) PXRD patterns of MEL (blue), 4DMP (green), MEL-4DMP salt (orange) and simulated pattern from the crystal structure (red) and (c) PXRD patterns of MEL (blue), PPZ (green), MEL -PPZ salt (orange) and simulated pattern from the crystal structure (red).

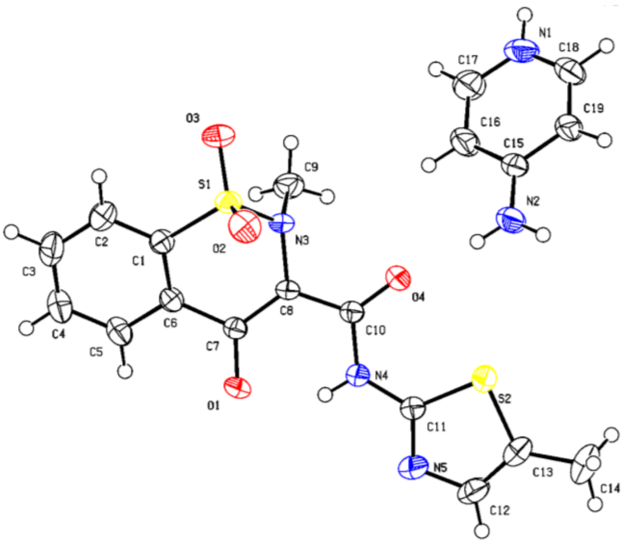

(a)

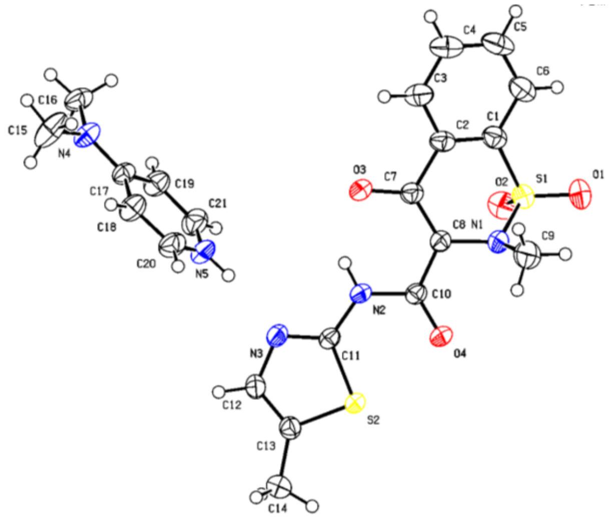

(b)

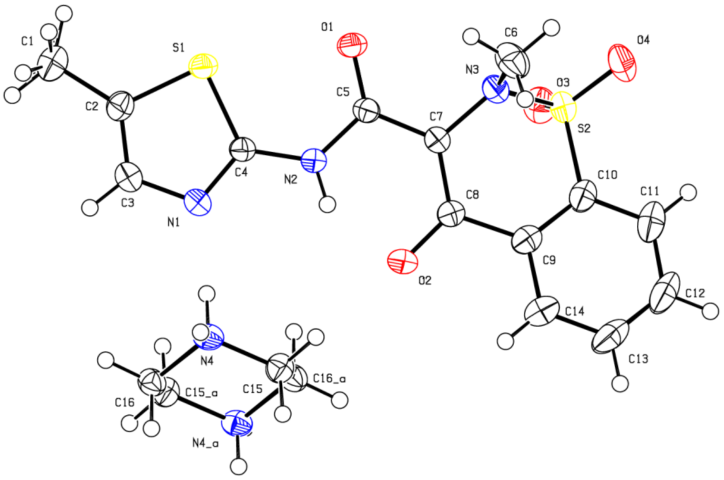

(c)

Figure S13. Ellipsoid plot of (a) MEL-4AP, (b) MEL-4DMP and (c) MEL-PPZ.

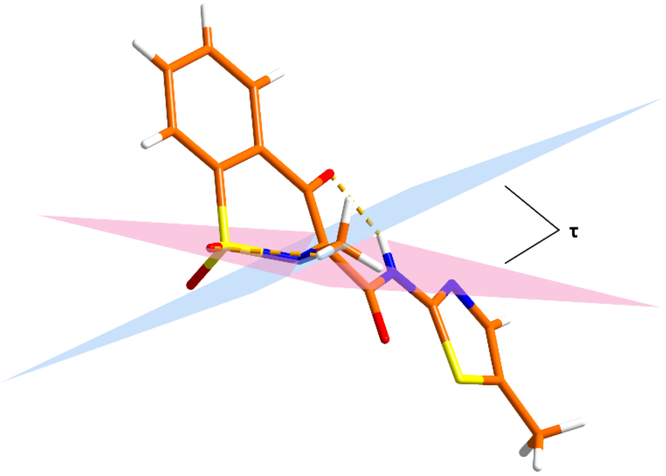

Solid	τ (°)
MEL-4AP	45.4
MEL-4DMP	45.6
MEL-PPZ	42.9

Figure S14. The angle (τ) formed between the methyl hydrogen and the plane of a sp^2 oxygen in MEL^- anion in MEL-4AP, MEL-4DMP and MEL-PPZ.

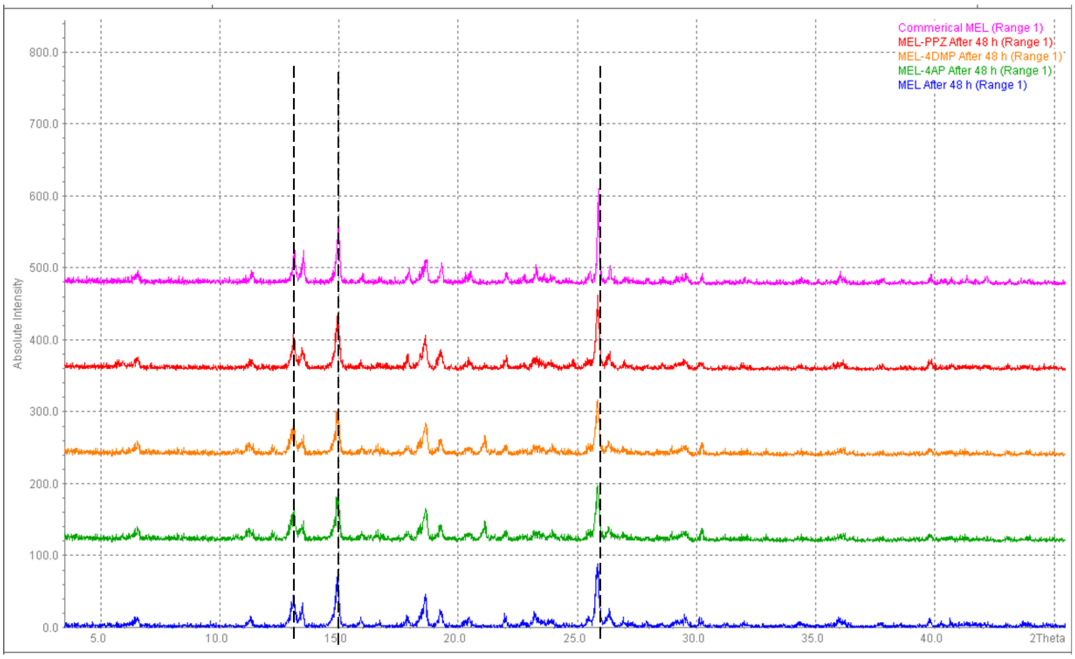

Figure S15. PXRD patterns of residual solids of MEL (blue), MEL-4AP (green), MEL-4DMP (orange), MEL-PPZ (red) after solubility experiments and the experimental PXRD patterns with commercial MEL (pink).

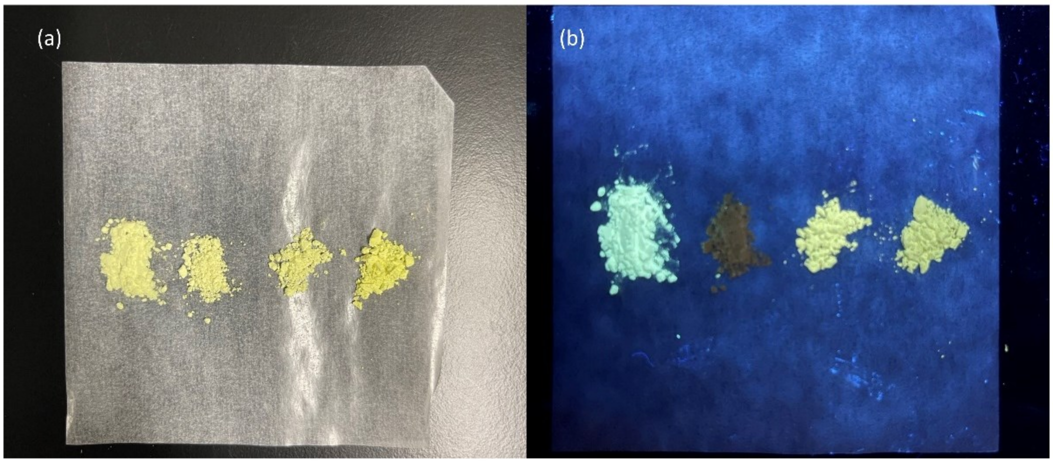

Figure S16. Photographs of MEL solids (from left to right: MEL, MEL-4AP, MEL-4DMP and MEL-PPZ): (a) powder samples under daylight; (b) powder samples under UV (365 nm) lamp.

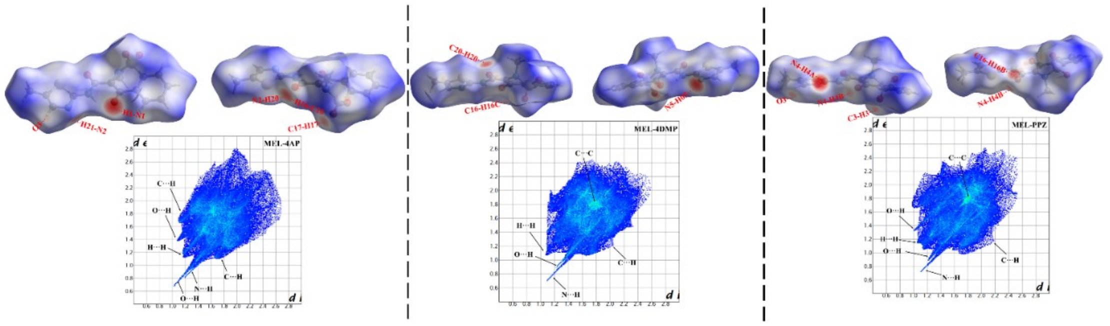

Figure S17. 3D d_{norm} surfaces and 2D fingerprint plots of MEL in MEL-4AP (left), MEL-4DMP (middle) and MEL-PPZ (right).

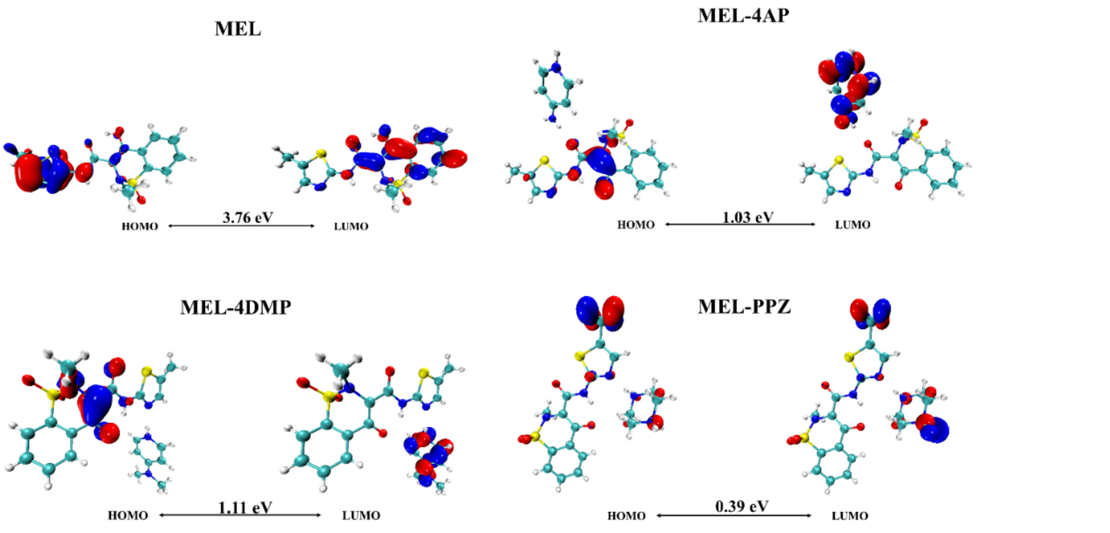

Figure S18. Molecular orbital plots of the HOMOs and LUMOs of MEL, MEL-4AP, MEL-4DMP and MEL-PPZ.

Table S5 Hydrogen bond and π–π interaction geometries (Å, °) in the MEL-4AP salt.

	Type	D-H···A	D-H	H···A	D···A	D-H···A	ARU (J)
1		N1-H1···O1	0.87(2)	1.835(19)	2.690(2)	166(3)	3445.01
2		N2-H20···O4	0.87(3)	2.07(3)	2.892(3)	157(2)	1555.01
3		N2-H21···N5	0.86(3)	2.17(3)	3.019(4)	168(2)	4464.01
4	Intra	N4-H22···O1	0.83(2)	1.88(3)	2.591(3)	142.7(19)	
5	Intra	C5-H5···O1	0.94	2.49	2.804(3)	100	
6		C12-H12···O3	0.93	2.57	3.259(3)	131	3455.01
7		C16-H16···O4	0.96	2.52	3.274(3)	135	1555.01
8		C17-H17···O2	0.94	2.37	3.290(4)	168	2565.01
9	Intra	C9-H24···O3	0.95	2.5	2.852(3)	102	

Cg(I)	Cg(J)	Cg-Cg	Interplanar distance	Dihedral Angle	Beta	ARU (J)
3	5	4.2197(16)	2.8815(10)	20.89(13)	27.2	2665.02

[a] Symmetry codes: [3445.] = -1/2+x, -1/2+y, z; [4464.] = -1/2+x, 3/2-y, -1/2+z; [3455.] = -1/2+x, 1/2+y, z; [2565.] = x, 1-y, 1/2+z; [2665.] = 1+x, 1-y, 1/2+z; [2464.] = -1+x, 1-y, -1/2+z. Cg3 represents the centroid of C1–C6 and Cg5 represents the centroid of N1, C15–C19. Cg(I) = plane number I; Cg–Cg = distance between ring centroids (Ang.). Beta is the displacement angle between the ring normal of plane I and the centroid vector.

Table S6 Hydrogen bond and π–π interaction geometries (Å, °) in the MEL-4DMP salt.

	Type	D-H···A	D-H	H···A	D···A	D-H···A	ARU (J)
1		N5-H05···N3	0.86	1.94	2.794(2)	172	[1555.01]
2	Intra	N2-H2···O3	0.86	1.86	2.5810(18)	140	
3	Intra	C9-H9A···O1	0.96	2.43	2.865(3)	107	
4		C15-H15A···O3	0.96	2.41	3.352(2)	169	[2766.01]
5		C16-H16A···O3	0.96	2.52	3.454(2)	163	[2766.01]
6		C16-H16C···O2	0.96	2.53	3.241(2)	131	[2666.01]
7		C20-H20···O4	0.93	2.26	3.162(2)	165	[2676.01]

Cg(I)	Cg(J)	Cg-Cg	Interplanar distance	Dihedral Angle	Beta	ARU (J)
3	3	3.7999(12)	3.4665(7)	0.00(9)	24.2	[2667.01]

[a] Symmetry codes: [2676.] = 1-x, 2-y, 1-z; [2666.] = 1-x, 1-y, 1-z; [2766.] = 2-x, 1-y, 1-z; [2667.] = 1-x, 1-y, 2-z. Cg3 is the centroid of C1-C6. Cg(I) = plane number I; Cg–Cg = distance between ring centroids (Ang.). Beta is the displacement angle between the ring normal of plane I and the centroid vector.

Table S7 Hydrogen bond and π–π interaction geometries (Å, °) in the MEL-PPZ salt.

	Type	D-H···A	D-H	H···A	D···A	D-H···A	ARU (J)
1	Intra	N2-H2···O2	0.86	1.77	2.520(4)	144	
2		N4-H4A···N1	0.89	1.96	2.847(4)	175	[1555.01]
3		N4-H4B···O1	0.89	2.22	2.928(4)	136	[1455.01]
4		N4-H4B···S1	0.89	2.83	3.314(3)	116	[3667.01]
5		N4-H4B···O1	0.89	2.32	2.969(4)	129	[3667.01]
6		C3-H3···O3	0.93	2.46	3.276(4)	146	[3666.01]
7	Intra	C6-H6A···O4	0.96	2.38	2.817(5)	107	

Cg(I)	Cg(J)	Cg-Cg	Interplanar distance	Dihedral Angle	Beta	ARU (J)
1	1	4.276(3)	3.3710(11)	0.00(13)	38	[3667.01]

[a] Symmetry codes: [3666.] = 1-x, 1-y, 1-z; [3667.] = 1-x, 1-y, 2-z; [1455.] = -1+x, y, z. Cg1 is the centroid of S1, C2–C4, N1. Cg(I) = plane number I; Cg–Cg = distance between ring centroids (Ang.). Beta is the displacement angle between the ring normal of plane I and the centroid vector.

Table S8 Summary of the various contact contributions to the MEL Hirshfeld surface area in pure MEL and its salts.

	O-H	H-H	C-H	C-O	C-C	N-H	O-N	N-C	N-N	H-S	O-O	S-C	S-N	S-O	S-S
**MEL**	28.9	35.1	9.9	2.2	4.6	5.1	0.9	4.6	0	7.2	0	0	0	1.3	0.2
**MEL-4AP**	26.5	32	20.8	1.1	1.9	7.5	0.1	0.8	0	5.7	0.7	1.6	0.7	0.6	0
**MEL-4DMP**	27.8	36.6	17.6	0.7	3.3	5.8	0.3	1.2	0.6	4.8	0	0	0.1	0.7	0.5
**MEL-PPZ**	27.3	38.9	16.6	1.4	3.0	5.2	0.3	1.2	0.6	4.4	0	0	0.1	0.9	0
